# Global, Regional, and National Incidence and Mortality of Neonatal Preterm Birth, 1990-2019

**DOI:** 10.1001/jamapediatrics.2022.1622

**Published:** 2022-05-31

**Authors:** Guiying Cao, Jue Liu, Min Liu

**Affiliations:** 1 Department of Epidemiology and Biostatistics, School of Public Health, Peking University, Beijing, China

## Abstract

**Importance:**

Preterm birth complications are the leading cause of death in children younger than 5 years worldwide. Despite advancing knowledge of risk factors and mechanisms related to preterm labor, the preterm birth rate has risen in most industrialized countries. Moreover, the burden of neonatal preterm birth remains unclear across the world.

**Objective:**

To determine the trends in incidence and mortality of neonatal preterm birth at the global, regional, and national levels to quantify its burden from 1990 to 2019 using data from the 2019 Global Burden of Disease study.

**Design, Setting, and Participants:**

Annual incident cases, deaths, age-standardized incidence rates (ASIRs), and age-standardized mortality rates (ASMRs) of neonatal preterm birth between 1990 and 2019 were collected from the 2019 Global Burden of Disease study. The percentage of relative changes in incident cases and deaths as well as the estimated annual percentage changes (EAPCs) of ASIRs and ASMRs were calculated to quantify their temporal trends. Correlations of EAPC of ASIRs and ASMRs with sociodemographic index (SDI) and universal health coverage index were evaluated by Pearson correlation analyses.

**Exposures:**

Infants born alive before 37 completed weeks of gestation between 1990 and 2019.

**Main Outcomes and Measures:**

Incident cases, deaths, ASIRs, and ASMRs of neonatal preterm birth.

**Results:**

Globally, the incident cases of neonatal preterm birth decreased by 5.26% from 16.06 million in 1990 to 15.22 million in 2019, and the deaths decreased by 47.71% from 1.27 million in 1990 to 0.66 million in 2019. The overall ASIR (EAPC = −0.19 [95% CI, −0.27 to −0.11]) and ASMR (EAPC = −2.09 [95% CI, −1.99 to −2.20]) decreased in this period. The ASIR of neonatal preterm birth increased by a mean of 0.25% (95% CI, 0.13%-0.38%) in high-SDI regions from 1990 to 2019. The ASMR of neonatal preterm birth increased by a mean of 2.09% (95% CI, 1.99%-2.20%) in Southern Sub-Saharan Africa in this period. A positive correlation was observed between EAPC of ASIR and SDI or universal health coverage index in 2019, while a negative correlation was observed between EAPC in ASMR and SDI or universal health coverage index in 2019 at national levels.

**Conclusions and Relevance:**

Preterm birth remains a crucial issue in children worldwide, with an increasing trend in ASIR in high-SDI regions and in ASMR in Southern Sub-Saharan Africa between 1990 to 2019. Efforts to reduce both the incidence and mortality of preterm births are essential.

## Key Points

**Question:**

What is the burden of neonatal preterm birth at the global, regional, and national levels?

**Findings:**

In this cross-sectional study using data from the Global Burden of Disease study, incident cases and deaths of neonatal preterm birth, overall age-standardized incidence rates (ASIRs), and age-standardized mortality rates (ASMRs) of neonatal preterm birth decreased from 1990 to 2019; however, ASIRs and ASMRs increased in some regions with high sociodemographic index regions and in Southern Sub-Saharan Africa, respectively. There was a positive correlation between estimated annual percentage change of ASIR and sociodemographic index or universal health coverage index in 2019, while there was a negative correlation between estimated annual percentage change in ASMR and sociodemographic index or universal health coverage index in 2019 at the national level.

**Meaning:**

Preterm birth remains a crucial issue in children worldwide and discovery research into the underlying mechanisms of neonatal preterm birth and the development of innovative interventions is urgent.

## Teaser

This cross-sectional study uses data from the 2019 Global Burden of Disease study to determine the trends in incidence and mortality of neonatal preterm birth at the global, regional, and national levels to quantify its burden from 1990 to 2019.

## Introduction

Preterm birth is defined as infants born alive before 37 completed weeks of gestation by the World Health Organization (WHO).^1^ Globally, it is estimated that 14.84 million infants were born preterm in 2014, and this number is rising.^2^ The preterm birth rate is 10.6% worldwide, ranging from 8.7% to 13.4% of infants born across regions.^2^ In addition, the preterm birth rate varies significantly across countries, with an increasing trend in most industrialized countries.^3^ For example, the preterm birth rate in the US increased from 9.5% in 1981 to 12.7% in 2005.^3^ Preterm birth is truly a global problem, despite more than 60% of preterm births occuring in Africa and South Asia.^1^

Preterm infants are particularly vulnerable to complications due to impaired respiration, difficulty in feeding, poor body temperature regulation, and high risk of infection.^4^ Preterm birth complications are the leading cause of death in children younger than 5 years worldwide and were responsible for approximately 1 million deaths in 2015.^1^ Global efforts to further reduce mortality in children younger than 5 years demand urgent action based on the data of incidence and mortality of preterm birth at regional and national levels as well as their associated factors. To our knowledge, there is currently no study to systematically clarify both the incidence and mortality of neonatal preterm birth at the global, regional, and national levels, as well as their association with socioeconomic status. Therefore, we retrieved detailed data on the incidence and mortality of neonatal preterm birth and socioeconomic status from the 2019 Global Burden of Disease (GBD) study to determine the global incidence and mortality of neonatal preterm birth and their associations with socioeconomic status at the national level to provide a more comprehensive perspective to make global and regional targeted interventions and health care policies for the prevention and control of neonatal preterm birth.

## Methods

### Data Source

The 2019 GBD study modeled nonfatal disease burden using DisMod-MR version 2.1, a meta-analysis tool that uses a compartmental model structure with a series of differential equations that synthesize sparse and heterogeneous epidemiologic data for nonfatal diseases, including neonatal disorders.^5^ In addition, the GBD study used standardized tools to generate estimates for the incidence and mortality of most diseases by age, sex, location, and year.^6,7^ In the GBD study, preterm birth is defined as infants born alive before 37 weeks of pregnancy according to the WHO.^8,9^ The detailed methods of the modeling strategy and alternative approaches for estimating neonatal preterm birth have been reported in previous studies.^5,6,7^ The study did not involve human participants and/or animals; therefore, no ethics approval or informed consent was needed.

This study used data of annual incident cases, deaths, age-standardized incidence rates (ASIRs), and age-standardized mortality rates (ASMRs) of neonatal preterm birth from 1990 to 2019 by sex, age, and location, collected from the Global Health Data Exchange query tool.^10^ Data were available from a total of 204 countries and territories, and these were categorized into 5 regions in terms of sociodemographic index (SDI) and 21 GBD regions according to geographical contiguity. Data on sociodemographic states, including SDI and universal health coverage index (UHCI), in 204 countries and territories used in this study were also collected from the Global Health Data Exchange query tool.^9^

### SDI

The SDI is a composite indicator of development status strongly correlated with health outcomes.^9^ It is the geometric mean of 0 to 1 indices of lag distributed income per capita, mean years of schooling for individuals 15 years and older, and total fertility rate for individuals younger than 25 years. A location with an SDI of 0 indicates a theoretical minimum level of development status relevant to health outcomes, while a location with an SDI of 1 indicates a theoretical maximum level.^9^ The SDIs of 204 countries and territories in 2019 are shown in eTable 1 in the Supplement.

### UHCI

The UHCI developed following GBD 2019 comprises 23 indicators drawn across a range of health care service areas and is meant to represent health care needs over the life course.^11^ The indicators of UHCI involved either direct measures of intervention coverage (eg, antiretroviral therapy coverage) or outcome-based indicators, such as mortality-to-incidence ratios, to approximate access to quality care.^12^ The UHCI indicators are reported on a scale of 0 to 100.^12^ The UHCIs of 204 countries and territories in 2019 are shown in eTable 1 in the Supplement.

### Statistical Analysis

We calculated the percentage of relative changes in incident cases and deaths of neonatal preterm birth and the estimated annual percentage changes (EAPCs) of ASIRs and ASMRs to quantify the trends in incidence of neonatal preterm birth. To compare the incidence and mortality rates of neonatal preterm birth across different populations, the ASIRs and ASMRs were carried out by applying the age-specific rates for each location, sex, and year to a GBD world standard population to adjust for potential confounding of age structure.^13^ The percentage of relative changes in incident cases of neonatal preterm birth from 1990 to 2019 was calculated by the equation: percentage of relative changes = (incident cases in 2019 − incident cases in 1990 / incident cases in 1990) × 100%. The percentage of relative changes in deaths of neonatal preterm birth were calculated using a similar equation. The EAPC is a summary and widely used measure of the age-standardized rate trend over a specified time interval. A regression line was fitted to the natural logarithm of the age-standardized rate, ie, y = α + βx + ε, where y = ln (age-standardized rate) and x = calendar year. The EAPC was calculated as 100 × (e^β^ − 1) and its 95% CI was calculated to reflect the temporal trend in age-standardized rate. The trend in age-standardized rate is reflected in EAPC value and its 95% CI age-standardized rate is in an upward trend when the EAPC and the lower boundary of the 95% CI are positive; conversely, age-standardized rate is in a downward trend when EAPC and the upper boundary of the 95% CI are negative. We calculated the EAPCs of ASIR and ASMR of neonatal preterm birth to reflect their temporal trends.

Moreover, the correlations of EAPC of ASIR and ASMR with SDI values (2019) and UHCI (2019) in 204 countries and territories were evaluated by Pearson correlation analyses to define the potential factors affecting EAPC. The polynomial curves were also modeled. All analyses were conducted with SAS version 9.4 (SAS Institute) and Origin 2019b (OriginLab). A 2-tailed *P* value less than .05 was considered statistically significant.

## Results

### Global Trend in Incidence and Mortality of Neonatal Preterm Birth

Globally, the number of incident cases of neonatal preterm birth decreased by 5.26% from 16.06 million in 1990 to 15.22 million in 2019, and the number of deaths of neonatal preterm birth decreased by 47.71% from 1.27 million in 1990 to 0.66 million in 2019 (Table 1). The overall ASIR of neonatal preterm birth decreased in the same period (EAPC = −0.19 [95% CI, −0.27 to −0.11]) from 244.19 per 100 000 in 1990 to 234.96 per 100 000 in 2019 (Table 2). The ASMR of neonatal preterm birth decreased by a mean of 2.09% (95% CI, 1.99%-2.20%) per year in the same period (from 19.34 per 100 000 in 1990 to 10.24 per 100 000 in 2019) (Table 2).

**Table 1.** Incident Cases and Deaths of Neonatal Preterm Birth in 1990 and 2019 and Their Change Trends From 1990 to 2019

Characteristic	1990		2019		Relative change, 1990-2019	
	Incident cases, No. × 10^5^ (95% UI)	Deaths, No. × 10^3^ (95% UI)	Incident cases, No. × 10^5^ (95% UI)	Deaths, No. × 10^3^ (95% UI)	Incident cases, %	Deaths, %
Overall	160.62 (159.46-161.90)	1269.04 (1166.14-1383.98)	152.17 (151.11-153.20)	663.52 (560.96-788.95)	−5.26	−47.71
Sex						
Female	73.42 (72.63-74.30)	560.11 (511.83-613.97)	69.75 (68.97-70.43)	279.57 (237.91-327.32)	−5.00	−50.09
Male	87.19 (86.34-88.05)	708.93 (644.55-781.04)	82.42 (81.70-83.18)	383.95 (321.74-463.85)	−5.47	−45.84
SDI region						
Low	NA	250.36 (221.71-283.47)	NA	261.93 (214.37-321.60)	NA	4.62
Low-middle	NA	474.63 (426.84-532.36)	NA	237.06 (196.74-280.55)	NA	−50.05
Middle	NA	376.48 (348.51-406.02)	NA	124.81 (105.33-147.61)	NA	−66.85
Middle-high	NA	135.26 (122.63-148.39)	NA	27.98 (23.74-32.93)	NA	−79.31
High	9.72 (9.63-9.81)	31.69 (29.80-33.81)	9.11 (9.01-9.21)	11.31 (10.07-12.66)	−5.00	−64.31
GBD region						
Andean Latin America	1.10 (1.05-1.15)	10.93 (9.51-12.51)	1.08 (1.04-1.13)	4.08 (2.93-5.48)	−1.78	−62.67
Australasia	0.22 (0.21-0.23)	0.65 (0.60-0.71)	0.27 (0.26-0.29)	0.25 (0.20-0.31)	25.30	−61.37
Caribbean	1.30 (1.26-1.34)	6.07 (5.28-6.96)	1.23 (1.18-1.27)	3.84 (2.78-5.23)	−5.15	−36.68
Central Asia	1.40 (1.36-1.45)	9.34 (8.15-10.65)	1.26 (1.22-1.30)	5.66 (4.63-6.90)	−10.16	−39.43
Central Europe	1.21 (1.18-1.23)	7.81 (7.36-8.29)	0.81 (0.79-0.82)	1.21 (0.92-1.52)	−33.16	−84.55
Central Latin America	4.23 (4.15-4.31)	39.64 (34.90-44.39)	4.07 (3.99-4.15)	11.58 (8.85-14.60)	−3.71	−70.78
Central Sub-Saharan Africa	2.91 (2.79-3.03)	25.74 (19.70-32.96)	4.50 (4.30-4.70)	27.70 (20.75-36.42)	54.46	7.60
East Asia	19.06 (18.76-19.37)	165.47 (145.14-187.30)	9.89 (9.74-10.05)	23.27 (19.88-27.07)	−48.10	−85.94
Eastern Europe	2.13 (2.10-2.16)	8.55 (7.81-9.47)	1.77 (1.75-1.80)	1.52 (1.21-1.88)	−16.68	−82.22
Eastern Sub-Saharan Africa	12.35 (12.16-12.54)	74.62 (64.54-85.04)	17.09 (16.83-17.37)	67.22 (51.93-87.25)	38.39	−9.92
High-income Asia Pacific	1.13 (1.10-1.17)	2.81 (2.50-3.26)	0.88 (0.86-0.90)	0.49 (0.42-0.56)	−22.58	−82.52
High-income North America	4.74 (4.70-4.78)	13.01 (12.37-13.74)	4.72 (4.65-4.78)	6.84 (6.21-7.58)	−0.47	−47.40
North Africa and Middle East	15.07 (14.77-15.41)	178.79 (152.22-212.21)	16.57 (16.24-16.92)	59.66 (47.21-74.22)	9.91	−66.63
Oceania	0.22 (0.21-0.23)	1.47 (1.08-1.90)	0.40 (0.37-0.42)	2.36 (1.57-3.44)	80.72	60.39
South Asia	58.10 (57.12-59.18)	438.60 (381.57-506.46)	46.85 (45.96-47.71)	233.42 (193.68-282.73)	−19.37	−46.78
Southeast Asia	12.77 (12.56-12.98)	119.38 (106.73-134.46)	9.63 (9.50-9.78)	44.54 (35.85-54.34)	−24.56	−62.69
Southern Latin America	0.57 (0.51-0.63)	7.36 (6.81-7.89)	0.55 (0.53-0.58)	2.31 (1.75-2.96)	−2.63	−68.64
Southern Sub-Saharan Africa	2.13 (2.09-2.17)	10.19 (8.54-11.99)	2.22 (2.17-2.26)	11.11 (8.63-14.66)	4.06	9.00
Tropical Latin America	4.14 (4.06-4.22)	47.02 (40.98-54.29)	3.71 (3.65-3.77)	11.31 (8.98-13.98)	−10.38	−75.95
Western Europe	3.45 (3.38-3.52)	9.91 (9.44-10.60)	3.19 (3.12-3.27)	3.21 (2.69-3.80)	−7.44	−67.65
Western Sub-Saharan Africa	12.40 (12.27-12.54)	91.68 (78.21-105.92)	21.50 (21.23-21.78)	141.95 (114.66-175.77)	73.32	54.83

Abbreviations: GBD, Global Burden of Disease; NA, not applicable; SDI, sociodemographic index; UI, uncertainty interval.

**Table 2.** ASIRs and ASMRs of Neonatal Preterm Birth in 1990 and 2019 and Their Change Trends From 1990 to 2019

Characteristic	No. (95% UI)				No. (95% CI)	
	1990		2019		1990-2019	
	ASIR per 100 000	ASMR per 100 000	ASIR per 100 000	ASMR per 100 000	EAPC of ASIR	EAPC of ASMR
Overall	244.19 (242.43 to 246.14)	19.34 (17.77 to 21.09)	234.96 (233.32 to 236.54)	10.24 (8.66 to 12.18)	−0.19 (−0.27 to −0.11)	−2.09 (−2.20 to −1.99)
Sex						
Female	230.89 (228.39 to 233.63)	17.65 (16.13 to 19.34)	223.07 (220.56 to 225.23)	8.94 (7.60 to 10.46)	−0.17 (−0.25 to −0.09)	−2.27 (−2.37 to −2.17)
Male	256.64 (254.13 to 259.16)	20.92 (19.02 to 23.05)	246.06 (243.90 to 248.33)	11.46 (9.60 to 13.84)	−0.20 (−0.29 to −0.12)	−1.96 (−2.06 to −1.86)
SDI region						
Low	323.61 (319.41 to 328.41)	22.25 (19.71 to 25.19)	294.30 (290.56 to 297.85)	14.53 (11.89 to 17.83)	−0.32 (−0.39 to −0.25)	−1.32 (−1.38 to −1.26)
Low-middle	302.97 (298.14 to 308.52)	26.25 (23.62 to 29.45)	255.10 (250.61 to 259.26)	13.96 (11.58 to 16.52)	−0.63 (−0.72 to −0.55)	−1.94 (−2.06 to −1.82)
Middle	202.36 (200.41 to 204.43)	18.23 (16.88 to 19.66)	194.49 (192.43 to 196.65)	7.26 (6.13 to 8.58)	−0.17 (−0.20 to −0.14)	−3.02 (−3.21 to −2.83)
Middle-high	174.85 (172.29 to 177.46)	13.58 (12.32 to 14.91)	173.43 (171.06 to 175.93)	3.69 (3.13 to 4.34)	−0.09 (−0.12 to −0.06)	−4.60 (−4.75 to −4.45)
High	171.30 (169.70 to 173.01)	5.57 (5.24 to 5.94)	183.62 (181.60 to 185.68)	2.27 (2.02 to 2.54)	0.25 (0.13 to 0.38)	−2.91 (−3.00 to −2.82)
GBD region						
Andean Latin America	190.40 (181.83 to 199.54)	18.96 (16.49 to 21.71)	171.80 (164.88 to 179.11)	6.47 (4.65 to 8.69)	−0.49 (−0.54 to −0.44)	−3.56 (−3.77 to −3.35)
Australasia	142.27 (135.63 to 149.31)	4.28 (3.96 to 4.68)	153.62 (144.93 to 162.26)	1.42 (1.13 to 1.73)	0.28 (0.17 to 0.39)	−3.05 (−3.44 to −2.66)
Caribbean	298.98 (290.28 to 307.94)	14.00 (12.19 to 16.05)	314.89 (303.19 to 326.29)	9.84 (7.11 to 13.39)	0.12 (0.08 to 0.17)	−1.08 (−1.18 to −0.98)
Central Asia	149.96 (144.96 to 154.52)	9.98 (8.71 to 11.38)	139.38 (134.69 to 144.10)	6.24 (5.11 to 7.61)	−0.33 (−0.37 to −0.30)	−1.35 (−1.73 to −0.96)
Central Europe	150.55 (147.39 to 153.64)	9.67 (9.13 to 10.28)	156.82 (154.09 to 159.69)	2.33 (1.77 to 2.94)	0.12 (0.09 to 0.14)	−4.80 (−5.05 to −4.54)
Central Latin America	177.97 (174.66 to 181.34)	16.70 (14.71 to 18.70)	192.96 (188.98 to 196.81)	5.48 (4.19 to 6.90)	0.30 (0.26 to 0.34)	−3.82 (−3.99 to −3.64)
Central Sub-Saharan Africa	228.14 (218.60 to 237.69)	20.24 (15.48 to 25.90)	211.19 (201.75 to 220.77)	13.01 (9.75 to 17.12)	−0.23 (−0.28 to −0.17)	−1.25 (−1.42 to −1.08)
East Asia	158.41 (155.99 to 161.05)	13.74 (12.05 to 15.55)	133.45 (131.36 to 135.55)	3.12 (2.66 to 3.63)	−0.76 (−0.89 to −0.63)	−5.32 (−5.60 to −5.05)
Eastern Europe	151.65 (149.30 to 153.94)	6.06 (5.54 to 6.71)	163.86 (161.57 to 165.90)	1.40 (1.11 to 1.72)	0.36 (0.33 to 0.38)	−5.35 (−5.70 to −5.00)
Eastern Sub-Saharan Africa	290.05 (285.64 to 294.45)	17.63 (15.25 to 20.11)	253.36 (249.44 to 257.46)	9.98 (7.71 to 12.95)	−0.48 (−0.60 to −0.35)	−1.86 (−1.96 to −1.76)
High-income Asia Pacific	119.85 (116.45 to 123.67)	2.96 (2.62 to 3.43)	132.50 (129.47 to 135.80)	0.73 (0.64 to 0.83)	0.46 (0.36 to 0.56)	−4.27 (−4.53 to −4.02)
High-income North America	215.56 (213.75 to 217.53)	5.90 (5.61 to 6.24)	233.97 (230.81 to 237.18)	3.38 (3.07 to 3.75)	0.26 (0.07 to 0.46)	−1.62 (−1.74 to −1.50)
North Africa and Middle East	268.82 (263.45 to 274.73)	31.99 (27.24 to 37.99)	284.77 (279.15 to 290.74)	10.24 (8.10 to 12.73)	0.17 (0.15 to 0.20)	−3.72 (−3.83 to −3.61)
Oceania	205.92 (194.46 to 217.48)	13.87 (10.20 to 17.87)	201.21 (188.27 to 213.86)	12.01 (8.01 to 17.48)	−0.09 (−0.14 to −0.04)	−0.35 (−0.44 to −0.26)
South Asia	338.16 (332.46 to 344.46)	25.62 (22.31 to 29.57)	292.10 (286.55 to 297.51)	14.56 (12.08 to 17.64)	−0.52 (−0.60 to −0.45)	−1.64 (−1.77 to −1.50)
Southeast Asia	211.47 (208.08 to 214.93)	19.80 (17.70 to 22.30)	184.13 (181.59 to 186.93)	8.51 (6.85 to 10.38)	−0.48 (−0.49 to −0.46)	−2.84 (−2.93 to −2.74)
Southern Latin America	113.24 (102.07 to 125.58)	14.67 (13.56 to 15.71)	119.31 (113.65 to 124.75)	4.97 (3.77 to 6.37)	0.25 (0.22 to 0.28)	−3.50 (−3.63 to −3.37)
Southern Sub-Saharan Africa	289.10 (283.41 to 295.04)	13.85 (11.61 to 16.29)	278.40 (272.49 to 284.35)	13.95 (10.84 to 18.42)	−0.16 (−0.24 to −0.08)	0.62 (0.20 to 1.03)
Tropical Latin America	241.73 (237.41 to 246.47)	27.50 (23.97 to 31.76)	239.95 (236.52 to 243.71)	7.30 (5.79 to 9.02)	0.02 (−0.01 to 0.04)	−4.48 (−4.59 to −4.37)
Western Europe	154.83 (151.70 to 158.09)	4.43 (4.22 to 4.74)	154.27 (150.64 to 157.90)	1.54 (1.29 to 1.83)	−0.01 (−0.04 to 0.01)	−3.45 (−3.60 to −3.31)
Western Sub-Saharan Africa	290.02 (286.89 to 293.26)	21.51 (18.35 to 24.85)	274.32 (270.86 to 277.93)	18.14 (14.65 to 22.46)	−0.19 (−0.22 to −0.16)	−0.48 (−0.55 to −0.41)

Abbreviations: ASIR, age-standardized incidence rate; ASMR, age-standardized mortality rate; EAPC, estimated annual percentage change; GBD, Global Burden of Disease; SDI, sociodemographic index; UI, uncertainty interval.

### Regional Trend in Incidence and Mortality of Neonatal Preterm Birth

In high-SDI regions, the incident cases of neonatal preterm birth decreased by 5.00% from 1990 to 2019 (Table 1), whereas the ASIR of neonatal preterm birth increased in the same period (EAPC = 0.25 [95% CI, 0.13-0.38]) from 171.30 per 100 000 in 1990 to 183.62 per 100 000 in 2019 (Table 2; eFigure 1A in the Supplement). The ASIR of neonatal preterm birth decreased in low-, low-middle–, middle-, and middle-high–SDI regions (Table 2; eFigure 1A in the Supplement). The number of deaths of neonatal preterm birth decreased by more than 50% in low-middle–, middle-, middle-high–, and high-SDI regions but increased by 4.62% in low-SDI regions from 1990 to 2019 (Table 1). In low-SDI regions, the growing number of deaths of neonatal preterm birth was derived from the increased number of deaths in nearly neonates aged 0 to 6 days (eFigure 2 in the Supplement). The decreasing number of deaths of neonatal preterm birth in low-middle–, middle-, middle-high–, and high-SDI regions was due to the gradual decrease in the number of deaths in all neonates, especially in nearly neonates aged 0 to 6 days (eFigure 2 in the Supplement). Across 5 SDI regions, the deaths and ASMR of neonatal preterm birth decreased in all regions, with the largest decrease in both deaths (79.31%) and ASMR (EAPC = −4.60 [95% CI, −4.75 to −4.45]) in middle-high–SDI regions (Table 1, Table 2; eFigure 1B in the Supplement).

Across the 21 GBD regions, the incident cases of neonatal preterm birth decreased in 66.7% of the regions (14 GBD regions) and the deaths of neonatal preterm birth decreased in 81.0% of the regions (17 regions) from 1990 to 2019, with the largest decrease both in incident cases (−48.10%) and deaths (−85.94%) in East Asia (Table 1). The nearly neonates aged 0 to 6 days accounted for more than 85% of deaths of neonatal preterm birth globally and approximately 90% in Central Sub-Saharan Africa (93.57%) and Western Sub-Saharan Africa (90.37%) in 2019 (eFigure 3 in the Supplement). In addition, the proportions of deaths of neonatal preterm birth in 2019 were higher than 20% for late neonates aged 7 to 28 days in Central Europe (23.74%) and postneonates aged 29 to 364 days in high-income Asia Pacific (21.88%) (eFigure 3 in the Supplement). Oceania experienced the largest increase both in incident cases (80.72%) and deaths (60.39%) from 1990 to 2019 (Table 1). The Caribbean experienced the most severe threat of incidence of neonatal preterm birth, with approximately 3 neonatal preterm births of 1000 populations (ASIR: 314.89 per 100 000) in 2019, followed by South Asia (ASIR in 2019: 292.10 per 100 000). For the mortality of neonatal preterm birth, Western Sub-Saharan Africa experienced the most severe threat (ASMR: 18.14 per 100 000) in 2019, followed by South Asia (ASMR in 2019: 14.56 per 100 000). The trends in ASIRs of neonatal preterm birth were heterogeneous across the 21 GBD regions from 1990 to 2019, with the highest increasing trend in high-income Asia Pacific (EAPC = 0.46 [95% CI, 0.36-0.56]), Central Latin America (EAPC = 0.30 [95% CI, 0.26-0.34]), and Australasia (EAPC = 0.28 [95% CI, 0.17-0.39]) and was stable in Western Europe and Tropical Latin America (Table 2). Nearly half of the GBD regions had a decreasing trend in ASIRs of neonatal preterm birth, such as Andean Latin America, East Asia, and South Asia (Table 2). The ASMR of neonatal preterm birth decreased in all GBD regions except Southern Sub-Saharan Africa (EAPC = 0.62 [95% CI, 0.20-1.03]) from 1990 to 2019 (Table 2).

### National Trend in Incidence and Mortality of Neonatal Preterm Birth

**Figure 1.**
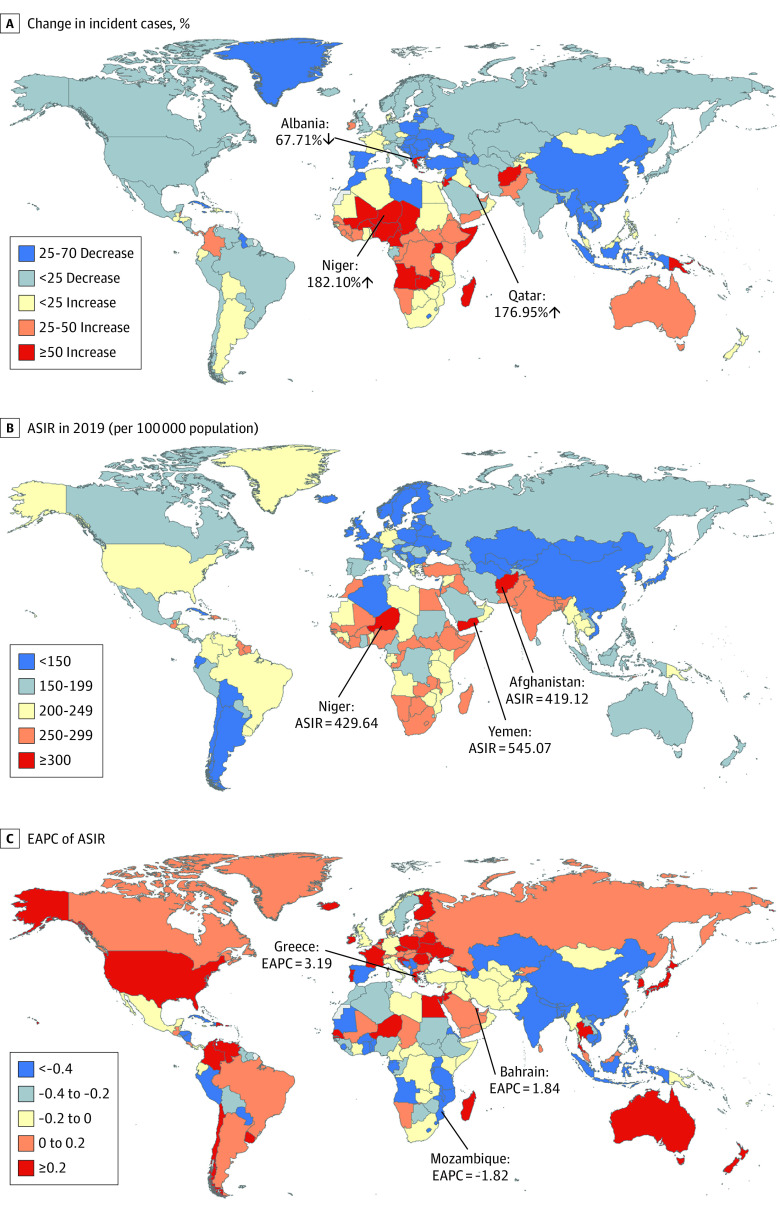
Global Trends in the Incidence of Neonatal Preterm Birth in 204 Countries and Territories The percentage of relative change in incident cases of neonatal preterm birth between 1990 and 2019 (A), age-standardized incidence rates (ASIRs) of neonatal preterm birth in 2019 (B), and estimated annual percentage changes (EAPCs) of ASIRs of neonatal preterm birth from 1990 to 2019 (C) are reported.

For 204 countries and territories, the absolute number of incident cases of neonatal preterm birth in India (3.10 million) and Pakistan (1.04 million) accounted for approximately one-third of the global incident cases (15.22 million) in 2019 (eTable 2 in the Supplement). The country with the most pronounced increase in incident cases of neonatal preterm birth was Niger (182.10%), followed by Qatar (176.95%) (eTable 2 in the Supplement; Figure 1A). The ASIR varies considerably across the world, with the largest ASIR in Yemen (545.07 per 100 000), followed by Niger (429.64 per 100 000) and Afghanistan (419.12 per 100 000) in 2019 (eTable 2 in the Supplement; Figure 1B). The ASIRs were deemed to be in a decreasing trend in 101 countries or territories, with the largest decrease in Mozambique (EAPC = −1.82 [95% CI, −2.05 to −1.58]) (eTable 2 in the Supplement; Figure 1C). The ASIRs were deemed to be in an increasing trend in 78 countries or territories, with the largest increase in Greece (EAPC = 3.91 [95% CI, 3.65-4.18]), followed by Bahrain (EAPC = 1.84 [95% CI, 1.69-1.99]) (eTable 2 in the Supplement; Figure 1C). The ASIRs remained stable in 25 countries or territories, such as Afghanistan, Cameroon, and Italy (eTable 2 in the Supplement).

**Figure 2.**
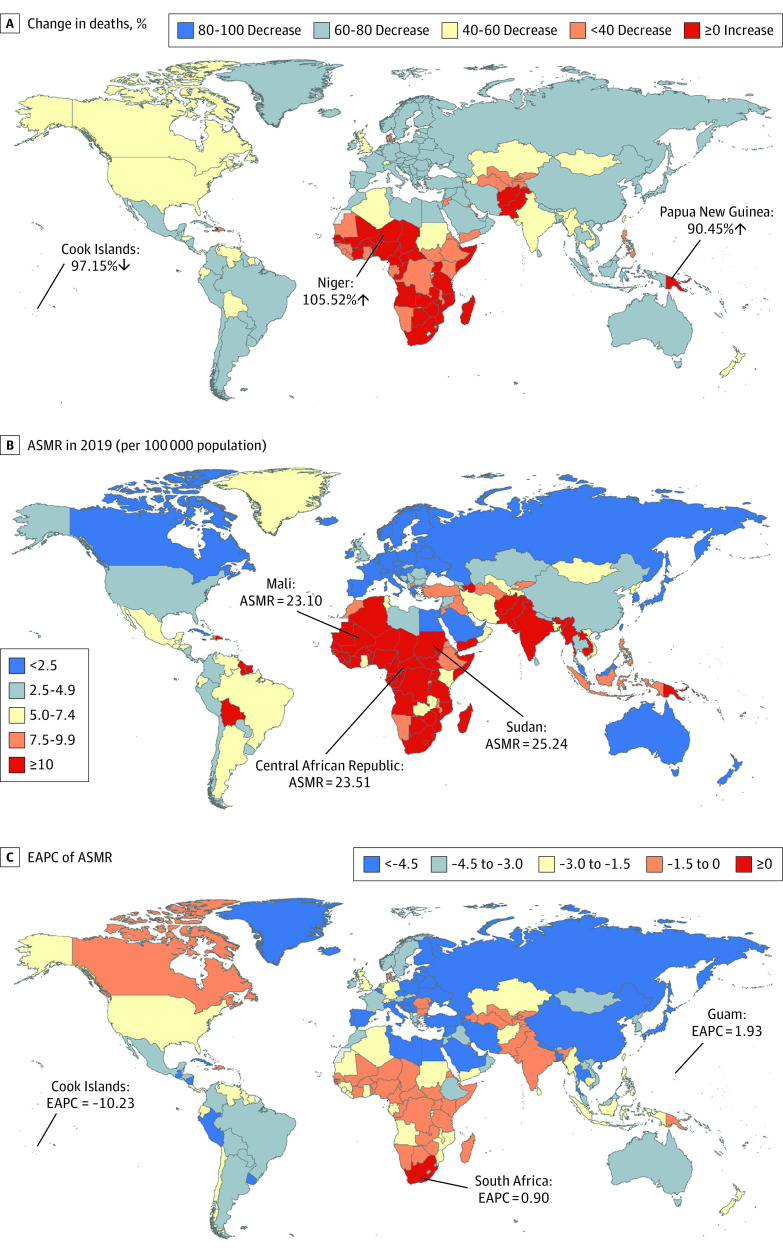
Global Trends in the Mortality of Neonatal Preterm Birth in 204 Countries and Territories The percentage of relative change in deaths of neonatal preterm birth between 1990 and 2019 (A), age-standardized mortality rates (ASMRs) of neonatal preterm birth in 2019 (B), and estimated annual percentage change (EAPCs) of ASMRs of neonatal preterm birth from 1990 to 2019 (C) are reported.

The most pronounced increase in deaths of neonatal preterm birth was observed in Niger (105.52%), followed by Papua New Guinea (90.45%), while the most pronounced decrease was detected in Cook Islands (97.15%) (eTable 3 in the Supplement; Figure 2A). The ASMR of neonatal preterm birth varied significantly across 204 countries and territories, with the largest ASMR in Sudan (ASMR: 25.54 per 100 000), Central African Republic (ASMR: 23.51 per 100 000), and Mali (ASMR: 23.10 per 100 000) in 2019 (eTable 3 in the Supplement; Figure 2B). The ASMRs were deemed to be in a decreasing trend in 186 countries or territories, with the largest decrease in Cook Islands (EAPC = −10.23 [95% CI, −11.01 to −9.45]) (eTable 3 in the Supplement; Figure 2C). The ASMRs were deemed to be increasing in 7 countries or territories, with the largest increase in Guam (EAPC = 1.93 [95% CI, 1.45-2.41]), followed by South Africa (EAPC = 0.90 [95% CI, 0.30-1.50]). The ASIRs remained stable in 11 countries or territories, such as Azerbaijan, Cote d’Ivoire, and Burkina Faso (eTable 3 in the Supplement).

### Correlations of EAPC of ASIR and ASMR With SDI and UHCI

**Figure 3.**
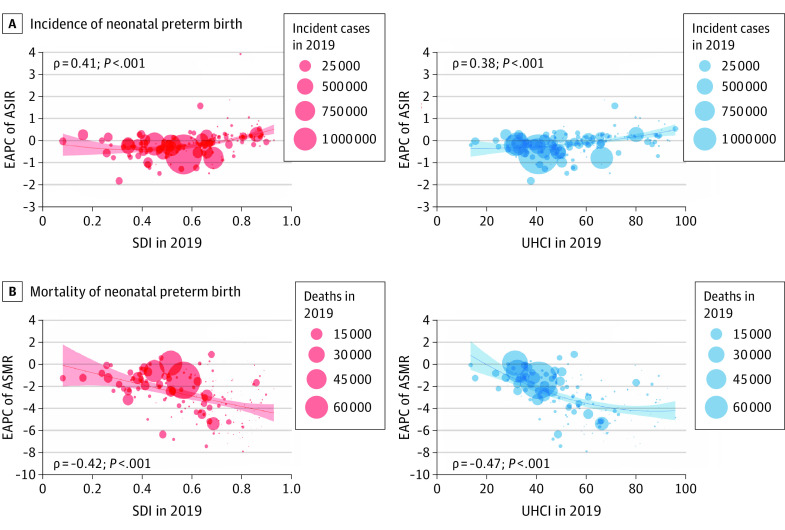
Estimated Annual Percentage Changes (EAPCs) of Age-Standardized Incidence Rates (ASIRs) and Age-Standardized Mortality Rates (ASMRs) in Neonatal Preterm Birth at the Country and Territorial Levels The incident cases and deaths of neonatal preterm birth from 204 countries and territories in 2019 are represented by circles. The size of the circles increased with the incident cases or deaths of neonatal preterm birth. The ρ indices and *P* values were derived from Pearson correlation analysis. SDI indicates sociodemographic index; UHCI, universal health coverage index.

A significant positive correlation was detected between EAPC of ASIR of neonatal preterm birth and SDI in 2019 (ρ = 0.41; *P* < .001) and UHCI in 2019 (ρ = 0.38; *P* < .001) (Figure 3A and B). Surprisingly, a significant negative correlation was detected between EAPC of ASMR of neonatal preterm birth and SDI in 2019 (ρ = −0.42; *P* < .001) and UHCI in 2019 (ρ = −0.47; *P* < .001) (Figure 3C and D).

## Discussion

To our knowledge, this is the first comprehensive assessment of the global landscape, long-term trends, and regional differences in the incidence and mortality of neonatal preterm birth, as well as the association with socioeconomic status at the national level, using data from the 2019 GBD study. In this study, we found that the global ASIR and ASMR of neonatal preterm birth decreased by a mean of 0.19% and 1.52% per year from 1990 to 2019, respectively. Meanwhile, the global absolute number of incident cases and deaths of neonatal preterm birth also decreased by 5.26% and 47.71% worldwide in this period, respectively. For SDI regions, the ASIRs of neonatal preterm birth increased in high-SDI regions by a mean of 0.25% per year from 1990 to 2019. The ASMR of neonatal preterm birth increased by a mean of 2.09% per year in Southern Sub-Saharan Africa in this period. Oceania experienced the largest increase both in the number of incident cases (82.72%) and deaths (60.39%) of neonatal preterm birth. Notably, we found a positive association of EAPC of ASIR with SDI or UHCI in 2019 and a negative association of EAPC of ASMR with SDI or UHCI in 2019 at the national level.

Preterm birth is an important perinatal health problem, contributing to increased mortality risk of children younger than 5 years directly and maternal mortality indirectly.^9,14,15^ Thus, reducing the incidence and mortality of preterm birth is significant. A better understanding of the incidence and mortality of preterm birth at the regional and national levels is needed to improve access to effective obstetric and neonatal care. In line with the decreasing trend in the global preterm birth rate,^3^ this current study found a decreasing trend in the ASIR and ASMR of neonatal preterm birth worldwide. This is largely attributed to improvements in maternal and newborn health care.^16,17^ Infant mortality from preterm birth can be reduced through interventions delivered to the birthing parent before or during pregnancy and to the preterm infant after birth.^4^ The progress in the frequency of the presence of a skilled birth attendant at birth in many countries presents a major opportunity to reduce intrapartum stillbirth and neonatal mortality.^16^ In addition, high-quality health care for female individuals is also essential in the prevention of neonatal preterm birth.^2^ Finally, improvements in nutritional status and medical interventions for reducing preterm birth are also beneficial for reducing the incidence and mortality of preterm birth.^16^

This study found that the ASIRs and ASMRs of neonatal preterm birth varied significantly across regions and nations. We found an increasing trend in the ASIR and a decreasing trend in the ASMR in high-SDI regions between 1990 and 2019, which was especially common in high-income countries, such as Greece, Bahrain, Japan, the UK, and the US. However, this study observed opposite trends in the ASIR and ASMR in some low-income countries: the ASMR of neonatal preterm birth increased but the ASIR of neonatal preterm birth decreased in Southern Sub-Saharan Africa. Similar to our findings, several previous studies reported that the incidence of preterm birth increased in high-income countries in the past 2 decades.^3,8,18,19^ Several possible factors contributing to but not completely explaining this increasing trend in the ASIR of neonatal preterm birth include increasing rates of multiple births, increases in the proportion of births among individuals older than 34 years, greater use of assisted reproduction techniques, and changes in clinical practices, such as greater use of elective cesarean delivery.^9,20,21^ For example, the increasing age of individuals giving birth in North America causes more maternal complications and cesarean deliveries and ultimately leads to an increased risk of preterm birth.^9^ In addition, the increasing use of ultrasonography rather than the date of the last menstrual period to estimate gestational age may have resulted in larger numbers of births being classified as preterm.^9^ In high-income countries, the reduction in mortality rates in infants who were born preterm has been driven largely by improved maternal and newborn health care.^4^ One previous study reported that preterm survival rates have increased in high-income countries, while preterm newborns still die because of a lack of adequate newborn care in many low-income and middle-income countries.^2^ Almost all births are attended by skilled staff and 50% of the neonates of less than 24 weeks’ gestation survive in high-income countries, whereas even the neonates older than 32 weeks’ gestation have only a 50% chance of survival owing to lack of available resources and/or low quality of specialized care in low-income countries.^22^ In high-income countries, administration of antenatal steroids is standard care for birthing parents with anticipated preterm labor, which has been verified to be very effective in preventing neonatal mortality; however, the coverage of antenatal steroid therapy remains low in low- and middle-income countries.^23^ In addition, intrauterine infection or lack of availability of drugs, such as tocolytic agents, might contribute to an increased risk of preterm birth and deaths of neonatal preterm birth in low-income countries.^9^ Thus, the development of interventions to reduce neonatal preterm birth is urgently needed for all countries, especially low-income countries where the incidence of neonatal preterm birth is high and the trend in mortality of neonatal preterm birth is increasing. These findings highlight the urgent need for discovery research into the underlying mechanisms of neonatal preterm birth and the development of innovative interventions.

### Limitations

This current study comprehensively assessed the global landscape, long-term trends, and regional differences in the incidence and mortality of neonatal preterm birth as well as the association with socioeconomic status using data from GBD estimates, which fill a gap where actual data on disease burden are sparse or unavailable. However, several limitations should be noted. First, the availability of data and the quality of available data limited the accuracy and robustness of the estimates of the incidence of mortality of neonatal preterm birth in the modeling, which might lead to bias when national surveillance and population-based studies were lacking. Second, data of gestational ages were not available in the GBD study; thus, we cannot further analyze the trends in incidence and mortality of neonatal preterm birth according to different gestational ages. Third, EAPC in ASIRs and ASMRs as well as the relative change in the number of incident cases and deaths were used to assess its long-term trends from 1990 to 2019, which might mask the recent short-term trends that reflected the effectiveness of the recent prevention interventions of preterm birth. Finally, the contribution of the causes of preterm birth is unknown globally.

## Conclusions

In summary, the global ASIR and ASMR of preterm birth gradually decreased from 1990 to 2019, while the ASIR increased in high SDI region and the ASMR increased in Southern Sub-Saharan Africa in this period. Preterm birth remains a crucial issue in children, both in high- and low-resource countries. Thus, efforts to reduce both the incidence and mortality of preterm births are essential worldwide.

## Notes

Supplement. eTable 1. The SDI and UHCI of 204 countries and territories in 2019 eTable 2. The incident cases and ASIRs of neonatal preterm birth in 1990 and 2019 and their change trends from 1990 to 2019 at national level eTable 3. The deaths and ASMRs of neonatal preterm birth in 1990 and 2019 and their change trends from 1990 to 2019 at national level eFigure 1. The ASIRs and ASMRs of neonatal preterm birth by SDI regions, from 1990 to 2019 eFigure 2. The deaths number of neonatal preterm birth by age group, by SDI regions, from 1990 to 2019 eFigure 3. The age distribution of deaths of neonatal preterm birth by GBD region in 1990 and 2019
