# Supplementary material for: Global, Regional, and National Incidence and Mortality of Neonatal Preterm Birth, 1990-2019
Source: JAMA Pediatr. 2022 May 31;176(8):787–96. doi: 10.1001/jamapediatrics.2022.1622 (PMC9157382; doi:10.1001/jamapediatrics.2022.1622)
Supplement: Supplement. — eTable 1. The SDI and UHCI of 204 countries and territories in 2019 eTable 2. The incident cases and ASIRs of neonatal preterm birth in 1990 and 2019 and their change trends from 1990 to 2019 at national level eTable 3. The deaths and ASMRs of neonatal preterm birth in 1990 and 2019 and their change trends from 1990 to 2019 at national level eFigure 1. The ASIRs and ASMRs of neonatal preterm birth by SDI regions, from 1990 to 2019 eFigure 2. The deaths number of neonatal preterm birth by age group, by SDI regions, from 1990 to 2019 eFigure 3. The age distribution of deaths of neonatal preterm birth by GBD region in 1990 and 2019 [file jamapediatr-e221622-s001.pdf]

Supplemental Online Content

Cao G, Liu J, Liu M. Global, regional, and national incidence and mortality of neonatal preterm birth, 1990-2019. *JAMA Pediatr*. Published online May 31, 2022. doi:10.1001/jamapediatrics.2022.1622

eTable 1. The SDI and UHCI of 204 countries and territories in 2019
eTable 2. The incident cases and ASIRs of neonatal preterm birth in 1990 and 2019 and their change trends from 1990 to 2019 at national level
eTable 3. The deaths and ASMRs of neonatal preterm birth in 1990 and 2019 and their change trends from 1990 to 2019 at national level
eFigure 1. The ASIRs and ASMRs of neonatal preterm birth by SDI regions, from 1990 to 2019
eFigure 2. The deaths number of neonatal preterm birth by age group, by SDI regions, from 1990 to 2019
eFigure 3. The age distribution of deaths of neonatal preterm birth by GBD region in 1990 and 2019

This supplemental material has been provided by the authors to give readers additional information about their work.

© 2022 Cao G et al. *JAMA Pediatrics.*

eTable 1. The SDI and UHCI of 204 countries and territories in 2019

Nation	SDI	UHCI
Afghanistan	0.343	39.295
Albania	0.681	69.626
Algeria	0.652	64.855
American Samoa	0.712	53.197
Andorra	0.894	91.748
Angola	0.470	39.158
Antigua and Barbuda	0.743	59.630
Argentina	0.708	61.158
Armenia	0.689	62.435
Australia	0.839	89.423
Austria	0.849	86.370
Azerbaijan	0.683	48.181
Bahamas	0.796	60.565
Bahrain	0.751	70.577
Bangladesh	0.483	53.883
Barbados	0.742	61.207
Belarus	0.745	70.462
Belgium	0.851	87.301
Belize	0.603	54.280
Benin	0.352	44.624
Bermuda	0.813	77.549
Bhutan	0.455	51.301
Bolivia	0.566	52.399
Bosnia and Herzegovina	0.718	64.184
Botswana	0.634	57.518
Brazil	0.640	64.828
Brunei	0.823	65.532
Bulgaria	0.764	62.557
Burkina Faso	0.257	41.797
Burundi	0.284	49.940
Cambodia	0.469	57.079
Cameroon	0.490	42.290
Canada	0.873	90.302
Cape Verde	0.525	62.188
Central African Republic	0.274	22.300
Chad	0.238	31.372
Chile	0.759	74.350
China	0.686	69.712
Colombia	0.633	74.397
Comoros	0.455	48.139
Congo	0.568	43.904

© 2022 Cao G et al. *JAMA Pediatrics.*

Cook Islands	0.764	62.228
Costa Rica	0.680	79.015
Cote d'Ivoire	0.408	43.040
Croatia	0.794	78.935
Cuba	0.668	72.592
Cyprus	0.841	79.602
Czech Republic	0.828	81.944
Democratic Republic of the Congo	0.382	45.169
Denmark	0.890	84.140
Djibouti	0.459	45.287
Dominica	0.729	51.809
Dominican Republic	0.592	52.500
Ecuador	0.640	64.454
Egypt	0.658	54.797
El Salvador	0.573	61.678
Equatorial Guinea	0.685	49.994
Eritrea	0.396	42.275
Estonia	0.835	82.039
Eswatini	0.577	53.397
Ethiopia	0.343	46.522
Federated States of Micronesia	0.580	34.474
Fiji	0.664	45.176
Finland	0.856	91.349
France	0.834	90.766
Gabon	0.656	53.050
Gambia	0.399	48.065
Georgia	0.702	55.953
Germany	0.898	86.249
Ghana	0.557	49.139
Greece	0.794	80.140
Greenland	0.761	68.725
Grenada	0.669	50.484
Guam	0.813	63.773
Guatemala	0.526	52.099
Guinea	0.325	32.335
Guinea-Bissau	0.355	35.707
Guyana	0.618	40.622
Haiti	0.432	35.812
Honduras	0.496	54.284
Hungary	0.791	72.028
Iceland	0.869	95.307
India	0.566	46.826
Indonesia	0.660	48.728

© 2022 Cao G et al. *JAMA Pediatrics.*

Iran	0.670	69.515
Iraq	0.671	57.725
Ireland	0.867	90.346
Israel	0.803	81.385
Italy	0.801	88.895
Jamaica	0.684	56.840
Japan	0.870	96.341
Jordan	0.731	69.967
Kazakhstan	0.723	59.237
Kenya	0.508	51.647
Kiribati	0.527	35.736
Kuwait	0.851	81.833
Kyrgyzstan	0.596	52.952
Lao	0.490	43.855
Latvia	0.820	69.788
Lebanon	0.708	74.533
Lesotho	0.507	38.737
Liberia	0.370	47.600
Libya	0.709	66.328
Lithuania	0.843	70.352
Luxembourg	0.895	91.455
Macedonia	0.744	60.746
Madagascar	0.396	39.691
Malawi	0.384	55.521
Malaysia	0.737	66.574
Maldives	0.562	66.857
Mali	0.263	40.661
Malta	0.801	82.881
Marshall Islands	0.544	44.004
Mauritania	0.496	53.278
Mauritius	0.705	55.809
Mexico	0.649	61.437
Moldova	0.696	62.194
Monaco	0.902	91.353
Mongolia	0.606	47.907
Montenegro	0.791	65.959
Morocco	0.548	58.032
Mozambique	0.307	44.044
Myanmar	0.521	46.954
Namibia	0.612	62.169
Nauru	0.618	42.002
Nepal	0.422	47.280
Netherlands	0.883	89.588

© 2022 Cao G et al. *JAMA Pediatrics.*

New Zealand	0.840	82.978
Nicaragua	0.517	57.159
Niger	0.162	35.026
Nigeria	0.515	38.339
Niue	0.711	49.010
North Korea	0.558	52.838
Northern Mariana Islands	0.771	60.411
Norway	0.913	94.241
Oman	0.783	71.221
Pakistan	0.449	39.168
Palau	0.738	45.067
Palestine	0.588	61.234
Panama	0.686	71.155
Papua New Guinea	0.394	37.767
Paraguay	0.638	63.351
Peru	0.648	75.759
Philippines	0.623	54.712
Poland	0.802	72.656
Portugal	0.743	83.533
Puerto Rico	0.814	75.531
Qatar	0.830	80.403
Romania	0.760	69.585
Russia	0.805	68.974
Rwanda	0.429	59.359
Saint Kitts and Nevis	0.746	52.885
Saint Lucia	0.670	59.140
Saint Vincent and the Grenadines	0.627	49.494
Samoa	0.641	49.796
San Marino	0.884	92.716
Sao Tome and Principe	0.502	54.755
Saudi Arabia	0.805	64.196
Senegal	0.389	49.610
Serbia	0.767	63.349
Seychelles	0.724	61.517
Sierra Leone	0.347	42.120
Singapore	0.861	92.440
Slovakia	0.812	77.983
Slovenia	0.840	89.834
Solomon Islands	0.407	39.333
Somalia	0.081	23.940
South Africa	0.678	59.727
South Korea	0.878	89.162
South Sudan	0.363	41.694

© 2022 Cao G et al. *JAMA Pediatrics.*

Spain	0.767	90.006
Sri Lanka	0.690	65.563
Sudan	0.515	51.835
Suriname	0.636	50.134
Sweden	0.872	90.361
Switzerland	0.929	93.498
Syrian Arab Republic	0.619	57.565
Taiwan (Province of China)	0.868	79.081
Tajikistan	0.539	47.878
Thailand	0.423	55.249
Timor-Leste	0.687	71.600
Togo	0.514	45.954
Tokelau	0.417	42.809
Tonga	0.626	52.643
Trinidad and Tobago	0.636	52.417
Tunisia	0.757	55.517
Turkey	0.672	68.106
Turkmenistan	0.748	69.210
Tuvalu	0.670	44.013
Uganda	0.589	39.570
Ukraine	0.404	52.748
United Arab Emirates	0.736	56.752
United Kingdom	0.880	63.357
Tanzania	0.847	87.900
United States of America	0.859	82.138
United States Virgin Islands	0.799	53.715
Uruguay	0.697	68.530
Uzbekistan	0.631	42.185
Vanuatu	0.485	34.084
Venezuela	0.607	60.968
Viet Nam	0.617	59.707
Yemen	0.412	49.046
Zambia	0.505	52.694
Zimbabwe	0.476	54.461

SDI: socio-demographic index; UHC: universal health coverage.

© 2022 Cao G et al. *JAMA Pediatrics.*

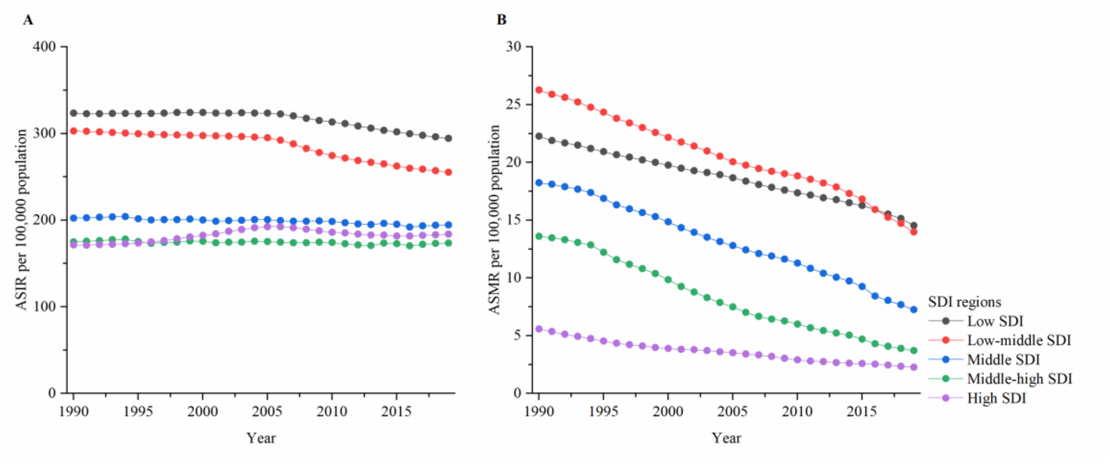

eFigure 1. The ASIRs and ASMRs of neonatal preterm birth by SDI regions, from 1990 to 2019.

Note: ASIR: age-standardized incidence rate; ASMR: age-standardized mortality rate; SDI: socio-demographic index.

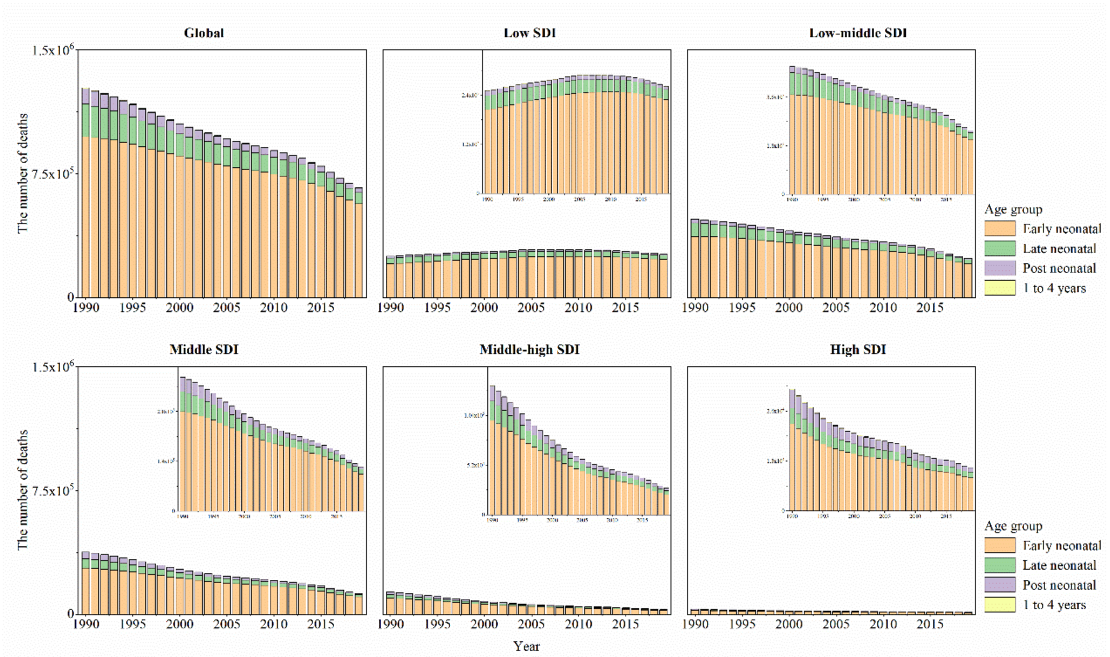

eFigure 2. The deaths number of neonatal preterm birth by age group, by SDI regions, from 1990 to 2019.

Note: SDI: socio-demographic index.

© 2022 Cao G et al. *JAMA Pediatrics.*

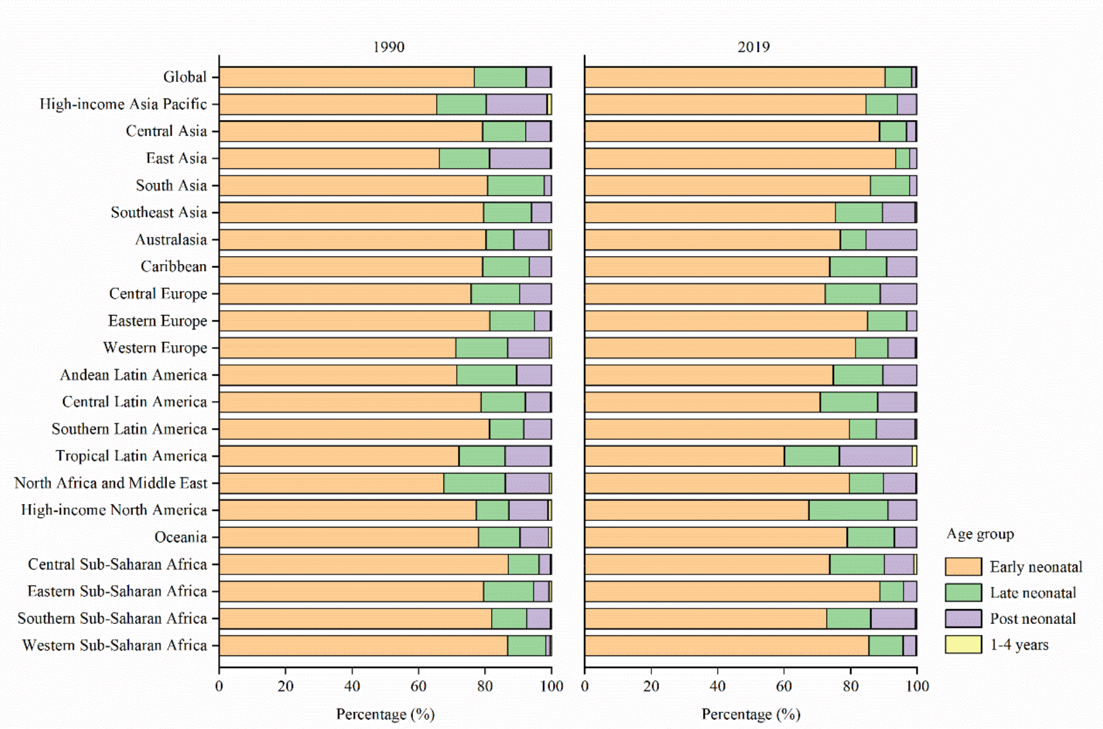

eFigure 3. The age distribution of deaths of neonatal preterm birth by GBD region in 1990 and 2019.

Note: GBD: Global Burden of Disease.

© 2022 Cao G et al. *JAMA Pediatrics.*

eTable 2. **The incident cases and ASIRs of neonatal preterm birth in 1990 and 2019 and their change trends from 1990 to 2019 at national level.**

Nation	1990		2019		1990-2019	
	Incident cases No. x 10^3 (95% UI)	ASIR per 100,000 No. (95% UI)	Incident cases No. x 10^3 (95% UI)	ASIR per 100,000 No. (95% UI)	Percentage of relative change in incident cases (%)	EAPC of ASIR No. (95% CI)
Afghanistan	112.52 (106.67, 117.95)	453.06 (429.53, 474.93)	297.92 (280.61, 315.54)	419.12 (394.77, 443.91)	164.78	-0.12 (-0.32, 0.08)
Albania	7.00 (6.52, 7.50)	170.16 (158.43, 182.38)	2.26 (2.09, 2.42)	134.49 (124.29, 143.96)	-67.71	-1.07 (-1.19, -0.96)
Algeria	54.97 (50.85, 59.28)	148.87 (137.70, 160.55)	60.60 (55.67, 65.87)	144.56 (132.80, 157.13)	10.24	-0.22 (-0.27, -0.17)
American Samoa	0.16 (0.15, 0.17)	196.08 (182.59, 210.19)	0.09 (0.09, 0.10)	183.21 (171.85, 195.48)	-42.36	-0.25 (-0.26, -0.23)
Andorra	0.04 (0.04, 0.04)	160.27 (147.69, 172.73)	0.04 (0.04, 0.05)	190.23 (177.59, 203.44)	5.98	0.70 (0.54, 0.87)
Angola	67.27 (63.19, 71.74)	276.58 (259.77, 294.96)	128.69 (120.60, 136.62)	242.98 (227.72, 257.97)	91.29	-0.40 (-0.45, -0.35)
Antigua and Barbuda	0.13 (0.12, 0.14)	205.79 (191.13, 220.55)	0.10 (0.10, 0.11)	219.95 (204.96, 235.86)	-21.75	0.26 (0.24, 0.28)
Argentina	33.60 (28.14, 39.56)	101.08 (84.66, 119.01)	34.85 (32.43, 37.30)	104.69 (97.42, 112.06)	3.71	0.17 (0.14, 0.20)
Armenia	6.57 (6.12, 7.02)	180.72 (168.13, 193.05)	3.28 (3.08, 3.46)	181.98 (171.10, 192.30)	-50.13	-0.04 (-0.13, 0.04)
Australia	17.87 (16.92, 18.92)	144.78 (137.08, 153.28)	22.58 (21.05, 24.08)	152.51 (142.19, 162.64)	26.35	0.20 (0.10, 0.30)
Austria	6.10 (5.62, 6.60)	136.42 (125.72, 147.45)	6.00 (5.57, 6.45)	141.29 (131.20, 151.87)	-1.66	0.11 (0.09, 0.12)
Azerbaijan	18.60 (17.37, 19.74)	204.30 (190.74, 216.85)	12.41 (11.70, 13.19)	180.45 (170.04, 191.73)	-33.26	-0.53 (-0.66, -0.40)
Bahamas	0.55 (0.51, 0.58)	211.32 (196.71, 225.76)	0.43 (0.41, 0.46)	218.02 (204.29, 232.46)	-20.71	0.03 (-0.01, 0.07)
Bahrain	1.04 (0.97, 1.12)	158.99 (148.54, 170.69)	1.49 (1.41, 1.59)	240.45 (227.21, 255.55)	43.09	1.84 (1.69, 1.99)
Bangladesh	740.51 (695.22, 782.46)	392.97 (368.94, 415.23)	446.40 (419.88, 471.49)	346.44 (325.86, 365.92)	-39.72	-0.50 (-0.60, -0.40)
Barbados	0.45 (0.42, 0.48)	225.10 (210.72, 239.40)	0.32 (0.30, 0.34)	229.68 (214.68, 245.12)	-30.27	0.05 (0.04, 0.06)
Belarus	7.10 (6.25, 7.98)	105.02 (92.36, 118.01)	5.58 (5.13, 6.03)	112.85 (103.75, 121.87)	-21.42	0.29 (0.27, 0.32)
Belgium	9.92 (9.22, 10.62)	164.69 (153.05, 176.26)	9.93 (9.21, 10.62)	168.04 (155.95, 179.83)	0.09	0.02 (-0.01, 0.05)
Belize	0.65 (0.60, 0.69)	228.81 (213.34, 244.07)	0.81 (0.76, 0.87)	221.50 (206.16, 237.91)	26.01	-0.02 (-0.08, 0.04)
Benin	29.74 (27.98, 31.63)	260.76 (245.31, 277.28)	53.16 (50.01, 56.22)	220.53 (207.48, 233.21)	78.74	-0.74 (-0.84, -0.65)
Bermuda	0.09 (0.09, 0.10)	215.45 (200.75, 230.61)	0.06 (0.05, 0.06)	235.67 (221.35, 249.73)	-37.34	0.35 (0.32, 0.38)
Bhutan	3.66 (3.40, 3.92)	346.81 (322.00, 371.24)	1.69 (1.59, 1.80)	263.53 (246.95, 280.47)	-53.79	-1.07 (-1.13, -1.02)
Bolivia	19.55 (18.22, 20.99)	167.81 (156.39, 180.18)	23.07 (21.16, 25.00)	149.35 (136.98, 161.88)	18	-0.35 (-0.40, -0.30)
Bosnia and Herzegovina	5.63 (5.28, 6.00)	172.14 (161.55, 183.46)	1.97 (1.84, 2.10)	153.03 (142.86, 163.09)	-64.97	-0.74 (-0.90, -0.58)
Botswana	7.10 (6.74, 7.51)	363.18 (344.74, 383.98)	7.68 (7.23, 8.15)	332.34 (313.21, 352.78)	8.05	-0.31 (-0.38, -0.24)
Brazil	401.64 (394.30, 409.87)	243.42 (238.97, 248.41)	360.10 (354.83, 365.73)	242.58 (239.03, 246.37)	-10.34	0.03 (0.01, 0.06)

© 2022 Cao G et al. *JAMA Pediatrics.*

Brunei	0.44 (0.41, 0.47)	130.22 (120.46, 140.56)		0.44 (0.41, 0.47)	143.13 (132.84, 154.46)		-0.27	0.36 (0.32, 0.39)
Bulgaria	7.05 (6.55, 7.60)	149.12 (138.52, 160.71)		4.30 (4.01, 4.59)	149.14 (139.23, 159.29)		-39	0.04 (0.00, 0.07)
Burkina Faso	75.25 (70.89, 79.56)	342.27 (322.45, 361.85)		143.66 (134.75, 152.47)	319.73 (299.90, 339.33)		90.91	-0.56 (-0.66, -0.46)
Burundi	35.06 (32.82, 37.42)	277.04 (259.30, 295.68)		52.15 (49.12, 55.37)	233.83 (220.26, 248.28)		48.73	-0.76 (-0.87, -0.65)
Cambodia	69.02 (64.65, 73.34)	329.09 (308.23, 349.66)		42.80 (40.04, 45.51)	243.78 (228.02, 259.21)		-37.99	-1.47 (-1.67, -1.28)
Cameroon	42.14 (39.60, 44.81)	189.09 (177.66, 201.03)		79.72 (74.25, 85.16)	184.49 (171.83, 197.09)		89.15	-0.07 (-0.14, 0.01)
Canada	30.31 (28.44, 32.23)	155.28 (145.72, 165.14)		29.36 (27.45, 31.25)	162.33 (151.75, 172.79)		-3.14	0.17 (0.15, 0.18)
Cape Verde	1.50 (1.40, 1.60)	265.87 (248.39, 283.86)		1.19 (1.11, 1.27)	230.82 (216.54, 246.17)		-20.86	-0.55 (-0.58, -0.52)
Central African Republic	17.67 (16.67, 18.84)	292.59 (276.04, 311.91)		26.19 (24.60, 27.73)	277.89 (261.04, 294.18)		48.22	-0.20 (-0.25, -0.15)
Chad	32.75 (30.61, 34.79)	215.24 (201.17, 228.69)		79.48 (74.28, 84.55)	210.56 (196.80, 223.99)		142.7	0.01 (-0.05, 0.08)
Chile	18.25 (17.04, 19.50)	127.74 (119.28, 136.51)		16.08 (15.02, 17.17)	147.73 (137.96, 157.69)		-11.88	0.69 (0.60, 0.78)
China	1815.02 (1785.89, 1846.71)	157.47 (154.95, 160.22)		945.30 (930.07, 961.07)	132.00 (129.87, 134.20)		-47.92	-0.78 (-0.92, -0.64)
Colombia	64.76 (60.35, 69.34)	144.76 (134.90, 154.99)		85.30 (80.48, 90.32)	224.46 (211.77, 237.66)		31.71	1.57 (1.42, 1.73)
Comoros	3.81 (3.61, 4.00)	403.21 (382.46, 423.41)		3.02 (2.85, 3.19)	381.58 (360.47, 403.14)		-20.72	-0.14 (-0.17, -0.10)
Congo	12.95 (12.11, 13.80)	270.92 (253.38, 288.68)		17.35 (16.20, 18.56)	252.02 (235.24, 269.61)		34.01	-0.21 (-0.25, -0.18)
Cook Islands	0.04 (0.04, 0.05)	193.23 (180.65, 206.50)		0.02 (0.02, 0.03)	185.46 (172.51, 198.80)		-45.53	-0.14 (-0.19, -0.09)
Costa Rica	6.21 (5.80, 6.67)	159.75 (149.07, 171.47)		5.14 (4.80, 5.54)	161.22 (150.54, 173.67)		-17.23	0.05 (0.03, 0.07)
Cote d'Ivoire	104.39 (98.29, 110.31)	382.03 (359.70, 403.67)		149.00 (139.92, 157.87)	352.53 (331.03, 373.50)		42.74	-0.13 (-0.23, -0.03)
Croatia	3.05 (2.80, 3.31)	113.68 (104.42, 123.57)		2.13 (1.97, 2.30)	124.11 (114.53, 134.03)		-30.06	0.35 (0.31, 0.39)
Cuba	16.45 (15.61, 17.30)	189.16 (179.46, 198.97)		6.70 (6.22, 7.18)	133.31 (123.69, 142.78)		-59.26	-1.47 (-1.72, -1.22)
Cyprus	0.92 (0.85, 0.99)	138.71 (127.99, 150.34)		1.04 (0.96, 1.12)	142.26 (131.14, 152.98)		13.23	0.15 (0.11, 0.20)
Czech Republic	8.59 (8.07, 9.14)	139.55 (131.15, 148.47)		8.97 (8.46, 9.46)	170.30 (160.67, 179.72)		4.4	1.05 (0.87, 1.24)
Democratic Republic of the Congo	184.32 (172.99, 195.51)	205.97 (193.31, 218.48)		268.30 (250.41, 287.44)	191.98 (179.18, 205.68)		45.56	-0.18 (-0.28, -0.08)
Denmark	3.60 (3.31, 3.90)	117.61 (108.00, 127.41)		3.73 (3.46, 4.02)	122.61 (113.75, 132.00)		3.76	0.19 (0.16, 0.21)
Djibouti	2.78 (2.60, 2.97)	262.91 (245.81, 280.18)		4.16 (3.90, 4.43)	251.17 (235.69, 267.48)		49.37	-0.30 (-0.37, -0.23)
Dominica	0.18 (0.17, 0.20)	225.06 (209.54, 241.67)		0.09 (0.08, 0.09)	214.57 (201.21, 228.02)		-53.53	-0.18 (-0.22, -0.14)
Dominican Republic	33.57 (31.27, 35.90)	293.14 (273.06, 313.46)		34.91 (32.65, 37.09)	319.20 (298.47, 339.07)		3.99	0.39 (0.34, 0.45)
Ecuador	22.05 (20.46, 23.93)	153.61 (142.53, 166.68)		24.71 (23.24, 26.27)	147.34 (138.56, 156.66)		12.04	-0.12 (-0.24, 0.00)
Egypt	308.23 (288.88, 327.51)	340.67 (319.28, 361.98)		358.64 (339.29, 377.49)	353.18 (334.13, 371.75)		16.35	0.20 (0.12, 0.28)
El Salvador	16.71 (15.56, 17.81)	212.66 (198.01, 226.66)		10.98 (10.31, 11.71)	203.48 (191.10, 217.11)		-34.31	-0.21 (-0.25, -0.17)

© 2022 Cao G et al. *JAMA Pediatrics.*

Equatorial Guinea	3.39 (3.18, 3.61)	309.45 (290.65, 329.33)		4.19 (3.91, 4.47)	226.03 (211.20, 241.58)		23.49	-1.25 (-1.41, -1.09)
Eritrea	22.64 (21.29, 24.16)	381.51 (358.79, 407.16)		28.44 (26.61, 30.29)	298.49 (279.25, 317.88)		25.61	-0.48 (-0.59, -0.37)
Estonia	1.14 (1.05, 1.22)	110.07 (102.22, 118.10)		0.75 (0.70, 0.80)	117.14 (109.10, 125.37)		-33.97	0.17 (0.09, 0.25)
Eswatini	3.17 (2.97, 3.37)	207.71 (194.31, 220.63)		2.56 (2.40, 2.71)	182.09 (170.53, 192.89)		-19.22	-0.46 (-0.49, -0.44)
Ethiopia	348.86 (337.68, 359.84)	294.26 (284.83, 303.52)		478.30 (462.80, 494.39)	273.26 (264.41, 282.45)		37.1	-0.24 (-0.45, -0.04)
Federated States of Micronesia	0.33 (0.31, 0.35)	197.42 (184.17, 212.59)		0.17 (0.16, 0.18)	182.34 (170.16, 195.76)		-48.2	-0.23 (-0.28, -0.19)
Fiji	1.63 (1.52, 1.74)	178.66 (166.71, 191.20)		1.45 (1.35, 1.56)	173.08 (161.03, 185.65)		-10.66	-0.12 (-0.14, -0.11)
Finland	3.36 (3.12, 3.61)	105.98 (98.28, 113.72)		2.97 (2.77, 3.17)	123.11 (114.84, 131.29)		-11.58	0.48 (0.41, 0.56)
France	33.09 (30.38, 35.84)	89.94 (82.57, 97.40)		33.16 (28.84, 37.69)	96.53 (83.93, 109.70)		0.22	0.23 (0.22, 0.25)
Gabon	5.52 (5.19, 5.87)	293.44 (276.22, 312.43)		4.95 (4.66, 5.23)	242.35 (228.09, 256.36)		-10.33	-0.79 (-0.96, -0.62)
Gambia	6.70 (6.31, 7.10)	290.92 (274.04, 308.41)		8.88 (8.31, 9.43)	260.86 (244.00, 276.91)		32.62	-0.40 (-0.41, -0.38)
Georgia	5.32 (4.96, 5.71)	132.78 (123.75, 142.47)		3.64 (3.46, 3.83)	166.32 (157.94, 174.82)		-31.57	0.64 (0.46, 0.83)
Germany	96.35 (90.89, 102.60)	224.78 (212.05, 239.36)		84.49 (79.60, 89.88)	229.59 (216.31, 244.26)		-12.31	-0.05 (-0.12, 0.02)
Ghana	71.26 (67.23, 75.92)	246.73 (232.77, 262.87)		74.32 (69.91, 78.82)	182.08 (171.26, 193.10)		4.3	-1.28 (-1.37, -1.18)
Greece	4.49 (4.12, 4.90)	89.70 (82.31, 97.99)		9.34 (8.80, 9.90)	225.63 (212.45, 239.02)		108.26	3.91 (3.65, 4.18)
Greenland	0.12 (0.11, 0.13)	212.26 (197.96, 226.49)		0.08 (0.07, 0.08)	206.84 (192.85, 221.22)		-35.08	0.00 (-0.11, 0.11)
Grenada	0.20 (0.18, 0.21)	208.69 (193.59, 225.10)		0.14 (0.13, 0.15)	215.88 (201.29, 231.34)		-27.55	0.12 (0.10, 0.13)
Guam	0.34 (0.32, 0.36)	187.90 (174.49, 201.51)		0.29 (0.26, 0.31)	182.60 (169.06, 195.75)		-15.99	-0.17 (-0.19, -0.14)
Guatemala	46.43 (43.79, 49.09)	287.41 (271.10, 303.87)		56.98 (53.31, 60.88)	288.20 (269.67, 307.94)		22.72	0.15 (0.02, 0.27)
Guinea	39.28 (36.76, 41.79)	260.00 (243.37, 276.65)		55.59 (52.23, 59.15)	242.54 (227.84, 258.04)		41.54	-0.28 (-0.35, -0.20)
Guinea-Bissau	8.65 (8.15, 9.16)	388.26 (365.60, 410.72)		10.68 (10.10, 11.22)	357.85 (338.52, 375.91)		23.36	-0.25 (-0.26, -0.24)
Guyana	4.30 (4.04, 4.55)	404.30 (380.38, 427.82)		2.53 (2.39, 2.68)	368.60 (348.06, 390.93)		-41.16	-0.32 (-0.36, -0.28)
Haiti	50.58 (47.89, 53.37)	428.03 (405.33, 451.66)		62.35 (58.71, 66.39)	393.89 (370.87, 419.40)		23.29	-0.41 (-0.46, -0.37)
Honduras	23.54 (21.79, 25.23)	273.60 (253.23, 293.26)		26.19 (24.44, 28.19)	232.32 (216.85, 250.12)		11.26	-0.75 (-0.87, -0.63)
Hungary	10.47 (9.84, 11.16)	175.73 (165.21, 187.39)		7.32 (6.91, 7.74)	184.32 (174.05, 194.95)		-30.09	0.17 (0.14, 0.19)
Iceland	0.30 (0.28, 0.33)	136.08 (126.35, 146.65)		0.30 (0.28, 0.32)	144.24 (134.35, 154.11)		-2.48	0.25 (0.21, 0.28)
India	4099.16 (4015.01, 4195.24)	325.58 (318.90, 333.21)		3103.78 (3034.23, 3172.23)	275.62 (269.45, 281.70)		-24.28	-0.61 (-0.71, -0.51)
Indonesia	460.42 (451.83, 468.88)	201.51 (197.75, 205.21)		316.90 (310.95, 322.78)	171.99 (168.77, 175.19)		-31.17	-0.51 (-0.58, -0.44)
Iran	161.70 (158.42, 164.82)	188.86 (185.02, 192.50)		123.82 (121.15, 126.49)	191.47 (187.35, 195.59)		-23.43	-0.10 (-0.15, -0.05)
Iraq	94.79 (88.55, 101.19)	282.17 (263.58, 301.23)		116.29 (108.54, 124.56)	252.90 (236.05, 270.88)		22.68	-0.13 (-0.24, -0.01)

© 2022 Cao G et al. *JAMA Pediatrics.*

Ireland	2.64 (2.43, 2.89)	102.21 (94.21, 111.88)		3.76 (3.50, 4.02)	129.25 (120.18, 138.15)		42.65	0.90 (0.67, 1.12)
Israel	6.46 (5.94, 6.99)	129.24 (118.86, 139.84)		12.87 (11.92, 13.87)	137.71 (127.55, 148.44)		99.07	0.23 (0.21, 0.25)
Italy	44.49 (43.37, 45.63)	165.89 (161.74, 170.17)		35.78 (34.92, 36.73)	167.43 (163.41, 171.88)		-19.56	-0.06 (-0.16, 0.03)
Jamaica	6.78 (6.35, 7.29)	252.04 (235.83, 270.84)		4.22 (3.95, 4.51)	243.97 (228.01, 260.25)		-37.71	-0.03 (-0.08, 0.02)
Japan	66.64 (65.40, 67.85)	112.32 (110.23, 114.37)		55.24 (54.26, 56.22)	127.16 (124.90, 129.40)		-17.1	0.54 (0.43, 0.65)
Jordan	17.22 (16.18, 18.28)	262.92 (247.07, 279.06)		33.51 (31.35, 35.74)	283.60 (265.35, 302.51)		94.59	0.31 (0.21, 0.42)
Kazakhstan	26.92 (24.98, 28.86)	158.16 (146.75, 169.56)		22.25 (20.62, 23.94)	131.34 (121.76, 141.33)		-17.36	-0.88 (-0.98, -0.77)
Kenya	112.38 (111.19, 113.64)	234.66 (232.19, 237.30)		144.92 (143.11, 146.57)	225.15 (222.34, 227.72)		28.96	-0.15 (-0.27, -0.03)
Kiribati	0.24 (0.23, 0.26)	198.12 (184.98, 211.49)		0.28 (0.26, 0.29)	190.09 (178.08, 202.73)		12.68	-0.05 (-0.09, -0.01)
Kuwait	3.95 (3.69, 4.22)	216.03 (201.89, 230.45)		6.40 (5.99, 6.82)	217.42 (203.43, 231.78)		61.85	0.18 (0.11, 0.25)
Kyrgyzstan	7.88 (7.27, 8.53)	130.23 (120.10, 141.07)		9.30 (8.61, 10.00)	133.25 (123.29, 143.35)		18.06	0.13 (0.10, 0.17)
Lao	16.98 (15.92, 18.14)	213.33 (200.00, 227.86)		15.51 (14.54, 16.47)	189.00 (177.15, 200.71)		-8.67	-0.26 (-0.44, -0.08)
Latvia	2.05 (1.88, 2.23)	116.48 (106.92, 126.83)		1.12 (1.03, 1.21)	120.81 (111.65, 130.70)		-45.47	0.17 (0.11, 0.23)
Lebanon	9.33 (8.71, 10.00)	177.36 (165.58, 189.97)		9.58 (8.91, 10.23)	189.38 (176.14, 202.23)		2.66	0.20 (0.08, 0.32)
Lesotho	9.07 (8.52, 9.60)	313.30 (294.21, 331.39)		5.69 (5.34, 6.04)	256.56 (241.05, 272.30)		-37.32	-0.73 (-0.76, -0.71)
Liberia	14.65 (13.81, 15.45)	380.99 (359.23, 401.87)		21.59 (20.36, 22.94)	335.68 (316.46, 356.59)		47.4	-0.26 (-0.39, -0.12)
Libya	15.33 (14.31, 16.41)	213.49 (199.34, 228.64)		7.95 (7.39, 8.55)	203.08 (188.59, 218.34)		-48.11	-0.08 (-0.20, 0.03)
Lithuania	3.24 (2.98, 3.51)	120.20 (110.35, 130.29)		1.58 (1.47, 1.69)	121.68 (113.18, 130.25)		-51.24	0.09 (0.03, 0.15)
Luxembourg	0.31 (0.29, 0.34)	129.64 (119.42, 140.88)		0.44 (0.41, 0.48)	139.01 (128.73, 149.96)		42.22	0.21 (0.17, 0.25)
Macedonia	2.24 (2.06, 2.43)	132.77 (122.24, 144.46)		1.44 (1.33, 1.55)	134.48 (124.47, 144.71)		-35.79	0.04 (-0.06, 0.14)
Madagascar	85.33 (80.53, 89.88)	334.70 (315.88, 352.57)		143.53 (134.85, 152.89)	350.25 (329.08, 373.09)		68.21	0.28 (0.18, 0.39)
Malawi	75.03 (71.39, 78.49)	336.49 (320.16, 352.01)		78.79 (74.50, 83.34)	299.99 (283.65, 317.31)		5.01	-0.69 (-0.84, -0.53)
Malaysia	43.43 (40.54, 46.37)	181.76 (169.65, 194.04)		49.02 (45.85, 52.24)	187.92 (175.78, 200.29)		12.86	0.16 (0.13, 0.19)
Maldives	1.06 (0.99, 1.13)	251.21 (234.45, 267.86)		0.97 (0.92, 1.03)	239.70 (226.12, 253.60)		-8.21	-0.22 (-0.32, -0.12)
Mali	78.40 (74.05, 82.92)	369.52 (348.99, 390.79)		172.58 (163.19, 182.61)	374.04 (353.67, 395.78)		120.12	0.16 (0.08, 0.24)
Malta	0.40 (0.38, 0.43)	148.92 (139.34, 158.97)		0.32 (0.30, 0.34)	156.26 (146.99, 165.68)		-19.69	0.27 (0.13, 0.41)
Marshall Islands	0.13 (0.12, 0.14)	177.60 (164.58, 190.00)		0.10 (0.10, 0.11)	172.70 (160.91, 184.20)		-20.99	-0.03 (-0.08, 0.02)
Mauritania	12.22 (11.46, 13.02)	286.25 (268.39, 304.95)		12.98 (12.14, 13.86)	247.46 (231.52, 264.19)		6.2	-0.56 (-0.61, -0.52)
Mauritius	1.93 (1.79, 2.08)	179.16 (165.60, 192.66)		1.15 (1.07, 1.24)	187.18 (173.98, 200.97)		-40.26	0.14 (0.13, 0.16)
Mexico	191.33 (187.09, 195.71)	160.69 (157.13, 164.37)		156.64 (153.33, 159.90)	155.73 (152.45, 158.98)		-18.13	-0.16 (-0.26, -0.07)

© 2022 Cao G et al. *JAMA Pediatrics.*

Moldova	4.95 (4.57, 5.34)	133.77 (123.45, 144.16)		2.10 (1.96, 2.25)	135.77 (126.68, 145.69)		-57.63	0.02 (-0.00, 0.05)
Monaco	0.02 (0.02, 0.02)	158.21 (146.86, 170.29)		0.02 (0.02, 0.02)	125.43 (115.34, 135.69)		-7.66	-0.84 (-0.89, -0.78)
Mongolia	4.80 (4.43, 5.23)	137.19 (126.66, 149.47)		5.20 (4.66, 5.76)	135.23 (121.08, 149.66)		8.35	-0.10 (-0.16, -0.04)
Montenegro	0.55 (0.51, 0.60)	115.44 (105.28, 125.21)		0.38 (0.35, 0.41)	119.05 (110.32, 128.31)		-30.96	0.08 (0.07, 0.10)
Morocco	108.81 (101.25, 116.80)	286.79 (266.88, 307.86)		74.12 (69.15, 79.53)	256.93 (239.70, 275.68)		-31.88	-0.32 (-0.36, -0.27)
Mozambique	95.78 (90.64, 100.75)	341.88 (323.51, 359.61)		112.71 (106.20, 119.59)	209.60 (197.51, 222.41)		17.67	-1.82 (-2.05, -1.58)
Myanmar	146.05 (136.72, 155.78)	229.70 (215.02, 245.01)		103.34 (96.54, 110.57)	206.59 (193.00, 221.04)		-29.24	-0.19 (-0.29, -0.09)
Namibia	7.66 (7.21, 8.11)	330.05 (310.71, 349.36)		9.77 (9.13, 10.42)	326.03 (304.88, 347.72)		27.53	0.07 (0.03, 0.10)
Nauru	0.04 (0.03, 0.04)	188.31 (174.97, 202.69)		0.03 (0.02, 0.03)	180.85 (167.96, 193.76)		-31.52	-0.20 (-0.24, -0.15)
Nepal	158.41 (149.06, 167.98)	405.91 (381.95, 430.43)		94.27 (89.04, 99.64)	319.82 (302.08, 338.04)		-40.49	-1.08 (-1.29, -0.88)
Netherlands	12.93 (11.94, 13.89)	135.79 (125.36, 145.87)		12.44 (11.49, 13.39)	144.46 (133.51, 155.50)		-3.81	0.29 (0.27, 0.31)
New Zealand	3.78 (3.57, 3.99)	131.51 (124.22, 138.73)		4.55 (4.32, 4.78)	159.42 (151.11, 167.39)		20.35	0.67 (0.51, 0.83)
Nicaragua	15.95 (14.79, 17.08)	231.37 (214.52, 247.87)		12.08 (11.01, 13.31)	192.04 (175.04, 211.71)		-24.28	-0.65 (-0.89, -0.40)
Niger	83.75 (79.71, 87.78)	393.19 (374.20, 412.10)		236.27 (223.21, 249.66)	429.64 (405.89, 453.99)		182.1	0.27 (0.09, 0.46)
Nigeria	535.55 (528.88, 541.92)	270.83 (267.46, 274.05)		910.26 (897.70, 923.77)	252.26 (248.78, 256.00)		69.97	-0.21 (-0.24, -0.17)
Niue	0.00 (0.00, 0.01)	179.40 (167.54, 192.44)		0.00 (0.00, 0.00)	179.79 (167.32, 192.31)		-52.94	-0.12 (-0.18, -0.06)
North Korea	62.10 (57.67, 66.64)	180.29 (167.40, 193.46)		28.68 (26.72, 30.82)	173.96 (162.06, 186.93)		-53.82	-0.21 (-0.28, -0.15)
Northern Mariana Islands	0.11 (0.11, 0.12)	183.71 (171.87, 196.29)		0.05 (0.04, 0.05)	193.39 (180.14, 206.69)		-59.66	0.15 (0.06, 0.25)
Norway	3.89 (3.81, 3.97)	134.15 (131.32, 137.08)		3.71 (3.63, 3.81)	134.01 (130.85, 137.32)		-4.49	-0.03 (-0.24, 0.19)
Oman	6.65 (6.17, 7.11)	209.58 (194.62, 223.95)		7.84 (7.31, 8.42)	209.45 (195.35, 224.80)		17.93	-0.01 (-0.06, 0.04)
Pakistan	808.18 (774.57, 842.31)	350.52 (335.94, 365.32)		1038.47 (994.69, 1081.86)	325.84 (312.10, 339.45)		28.49	-0.14 (-0.22, -0.07)
Palau	0.03 (0.03, 0.04)	187.75 (173.92, 201.47)		0.02 (0.02, 0.02)	188.61 (175.67, 203.05)		-49.03	-0.03 (-0.06, 0.01)
Palestine	9.15 (8.56, 9.76)	200.61 (187.78, 213.95)		11.52 (10.66, 12.32)	192.25 (177.90, 205.57)		25.96	-0.14 (-0.20, -0.07)
Panama	5.43 (5.08, 5.79)	191.59 (179.04, 204.18)		6.90 (6.47, 7.33)	187.63 (175.93, 199.55)		26.89	-0.05 (-0.09, -0.01)
Papua New Guinea	15.11 (13.99, 16.26)	211.81 (196.11, 227.94)		32.07 (29.69, 34.38)	204.53 (189.37, 219.29)		112.24	-0.14 (-0.19, -0.08)
Paraguay	12.04 (11.21, 12.83)	196.21 (182.65, 209.05)		10.64 (9.94, 11.33)	175.63 (164.00, 186.96)		-11.62	-0.44 (-0.55, -0.33)
Peru	68.37 (64.02, 72.95)	215.32 (201.62, 229.75)		60.24 (56.53, 63.87)	196.48 (184.39, 208.35)		-11.89	-0.56 (-0.70, -0.42)
Philippines	232.72 (229.29, 236.45)	234.77 (231.32, 238.54)		251.47 (247.68, 255.49)	198.09 (195.11, 201.26)		8.06	-0.53 (-0.60, -0.46)
Poland	36.13 (35.28, 37.10)	140.10 (136.80, 143.85)		26.17 (25.64, 26.71)	149.87 (146.85, 152.99)		-27.58	0.21 (0.18, 0.23)
Portugal	6.96 (6.43, 7.53)	125.99 (116.41, 136.29)		6.45 (6.00, 6.94)	167.89 (156.01, 180.52)		-7.3	1.03 (0.95, 1.11)

© 2022 Cao G et al. *JAMA Pediatrics.*

Puerto Rico	6.81 (6.35, 7.28)	216.13 (201.58, 230.78)		2.83 (2.65, 3.03)	233.48 (218.37, 249.85)		-58.42	0.32 (0.29, 0.35)
Qatar	1.37 (1.28, 1.45)	270.86 (254.41, 287.83)		3.78 (3.55, 4.01)	288.40 (271.00, 305.80)		176.95	0.27 (0.24, 0.31)
Romania	23.23 (21.65, 24.93)	162.81 (151.73, 174.73)		14.92 (14.04, 15.81)	178.47 (167.94, 189.06)		-35.76	0.24 (0.15, 0.33)
Russia	161.73 (160.22, 163.34)	174.22 (172.59, 175.95)		145.63 (143.91, 147.29)	181.44 (179.31, 183.52)		-9.96	0.18 (0.16, 0.21)
Rwanda	32.97 (30.87, 35.12)	216.59 (202.76, 230.73)		28.12 (26.29, 29.95)	166.48 (155.62, 177.31)		-14.71	-1.22 (-1.50, -0.95)
Saint Kitts and Nevis	0.09 (0.08, 0.10)	209.27 (195.24, 222.12)		0.07 (0.07, 0.08)	220.55 (206.37, 235.48)		-18.48	0.20 (0.19, 0.21)
Saint Lucia	0.40 (0.37, 0.43)	220.19 (204.96, 235.17)		0.19 (0.18, 0.20)	220.38 (205.13, 235.45)		-52.86	0.02 (0.01, 0.04)
Saint Vincent and the Grenadines	0.27 (0.26, 0.29)	215.51 (200.88, 229.57)		0.15 (0.14, 0.16)	209.96 (195.83, 224.10)		-44.38	-0.08 (-0.11, -0.05)
Samoa	0.32 (0.30, 0.34)	194.96 (181.14, 209.26)		0.35 (0.33, 0.38)	192.12 (179.01, 206.10)		10.15	0.02 (-0.02, 0.06)
San Marino	0.02 (0.02, 0.02)	128.32 (118.81, 138.45)		0.02 (0.02, 0.02)	141.54 (130.63, 151.86)		26.55	0.35 (0.34, 0.37)
Sao Tome and Principe	0.79 (0.74, 0.84)	353.08 (331.28, 375.52)		0.66 (0.62, 0.70)	293.13 (275.40, 312.74)		-16.32	-0.76 (-0.81, -0.71)
Saudi Arabia	41.24 (38.45, 44.15)	160.47 (149.60, 171.80)		35.31 (33.12, 37.76)	161.35 (151.34, 172.57)		-14.38	0.16 (0.04, 0.28)
Senegal	54.07 (51.07, 57.27)	329.85 (311.51, 349.34)		72.88 (69.28, 76.76)	328.22 (312.01, 345.71)		34.78	0.28 (0.16, 0.40)
Serbia	10.18 (9.39, 10.98)	159.30 (146.90, 171.80)		5.68 (5.29, 6.09)	143.26 (133.46, 153.64)		-44.21	-0.50 (-0.60, -0.40)
Seychelles	0.15 (0.14, 0.16)	182.47 (169.93, 194.71)		0.13 (0.13, 0.14)	187.47 (175.68, 199.82)		-9.6	0.13 (0.11, 0.15)
Sierra Leone	24.52 (23.01, 26.26)	325.51 (305.51, 348.71)		36.01 (33.69, 38.31)	269.66 (252.24, 286.88)		46.9	-0.66 (-0.74, -0.59)
Singapore	4.19 (3.93, 4.46)	170.81 (160.16, 181.86)		5.18 (4.86, 5.48)	188.30 (176.52, 199.27)		23.7	0.75 (0.43, 1.06)
Slovakia	5.41 (5.02, 5.83)	143.26 (132.77, 154.30)		4.00 (3.73, 4.28)	149.37 (139.15, 159.82)		-26.09	0.18 (0.15, 0.20)
Slovenia	1.45 (1.35, 1.57)	136.49 (126.42, 147.45)		1.33 (1.25, 1.42)	146.68 (137.27, 156.67)		-8.33	0.30 (0.27, 0.33)
Solomon Islands	1.41 (1.31, 1.50)	205.70 (191.16, 219.76)		1.92 (1.78, 2.05)	191.86 (178.50, 204.87)		36.19	-0.29 (-0.35, -0.24)
Somalia	46.03 (43.31, 48.81)	312.58 (294.09, 331.43)		120.59 (112.64, 129.18)	289.73 (270.63, 310.36)		161.97	-0.02 (-0.10, 0.06)
South Africa	139.20 (136.11, 142.61)	298.37 (291.75, 305.67)		146.08 (142.44, 149.61)	295.59 (288.21, 302.71)		4.94	-0.06 (-0.09, -0.03)
South Korea	42.03 (38.95, 45.07)	129.67 (120.19, 139.07)		26.85 (25.04, 28.73)	136.33 (127.12, 145.89)		-36.11	0.27 (0.17, 0.36)
South Sudan	35.15 (32.99, 37.42)	292.58 (274.58, 311.49)		46.23 (43.24, 49.45)	267.39 (250.11, 286.00)		31.54	-0.28 (-0.38, -0.18)
Spain	39.05 (37.27, 41.03)	204.86 (195.51, 215.22)		27.21 (25.45, 28.93)	154.32 (144.39, 164.10)		-30.34	-0.70 (-0.85, -0.54)
Sri Lanka	33.84 (31.61, 36.35)	197.47 (184.44, 212.13)		28.50 (26.39, 30.70)	199.92 (185.09, 215.36)		-15.77	0.08 (0.05, 0.11)
Sudan	82.07 (76.15, 88.09)	176.07 (163.36, 188.98)		90.23 (84.12, 96.44)	160.43 (149.57, 171.47)		9.95	-0.35 (-0.41, -0.29)
Suriname	1.29 (1.20, 1.38)	295.54 (276.18, 316.86)		1.18 (1.10, 1.26)	274.34 (256.12, 294.07)		-8.26	-0.28 (-0.33, -0.23)
Sweden	5.65 (5.27, 6.05)	94.32 (87.96, 101.01)		5.02 (4.66, 5.35)	87.86 (81.66, 93.80)		-11.22	-0.25 (-0.29, -0.20)
Switzerland	6.07 (5.68, 6.48)	151.64 (141.84, 161.99)		6.60 (6.15, 7.06)	154.90 (144.18, 165.51)		8.82	0.03 (0.00, 0.05)

© 2022 Cao G et al. *JAMA Pediatrics.*

Syrian Arab Republic	55.41 (51.62, 59.33)	229.58 (213.87, 245.80)		22.83 (21.26, 24.31)	203.98 (189.99, 217.24)		-58.81	-0.10 (-0.29, 0.09)
Taiwan (Province of China)	28.39 (26.87, 30.09)	179.11 (169.50, 189.80)		15.00 (14.03, 16.04)	176.57 (165.24, 188.85)		-47.18	0.17 (0.07, 0.28)
Tajikistan	21.24 (19.86, 22.54)	216.40 (202.36, 229.73)		23.28 (21.74, 24.77)	190.95 (178.29, 203.14)		9.64	-0.50 (-0.58, -0.43)
Thailand	166.71 (157.32, 176.31)	286.79 (270.63, 303.31)		204.77 (191.96, 217.59)	207.52 (194.54, 220.51)		22.83	-0.98 (-1.13, -0.84)
Timor-Leste	101.31 (94.70, 107.68)	199.21 (186.20, 211.73)		57.10 (53.67, 60.82)	203.27 (191.06, 216.52)		-43.64	0.22 (0.08, 0.35)
Togo	6.88 (6.48, 7.30)	402.06 (378.81, 426.93)		5.77 (5.43, 6.13)	313.81 (295.43, 333.38)		-16.09	-1.07 (-1.17, -0.96)
Tokelau	24.63 (23.12, 26.25)	314.92 (295.52, 335.61)		30.75 (28.83, 32.66)	272.16 (255.16, 289.04)		24.85	-0.53 (-0.58, -0.47)
Tonga	0.01 (0.01, 0.01)	196.44 (182.97, 210.65)		0.00 (0.00, 0.00)	178.32 (166.67, 190.18)		-43.25	-0.36 (-0.45, -0.27)
Trinidad and Tobago	0.23 (0.21, 0.24)	181.86 (168.82, 195.51)		0.19 (0.18, 0.21)	172.91 (160.48, 185.47)		-14.63	-0.23 (-0.28, -0.18)
Tunisia	2.30 (2.14, 2.45)	204.24 (190.57, 217.93)		1.57 (1.46, 1.68)	212.35 (197.96, 227.50)		-31.68	0.12 (0.11, 0.12)
Turkey	21.68 (20.14, 23.17)	190.81 (177.26, 203.95)		13.79 (12.88, 14.66)	171.94 (160.69, 182.84)		-36.4	-0.31 (-0.41, -0.22)
Turkmenistan	225.00 (210.43, 240.54)	285.21 (266.73, 304.91)		130.23 (121.65, 139.28)	277.14 (258.88, 296.40)		-42.12	-0.18 (-0.23, -0.13)
Tuvalu	6.76 (6.18, 7.35)	107.73 (98.46, 117.06)		5.81 (5.32, 6.29)	108.16 (99.06, 117.20)		-14.15	-0.06 (-0.11, -0.02)
Uganda	0.03 (0.02, 0.03)	190.86 (177.56, 204.16)		0.02 (0.02, 0.02)	178.79 (166.62, 190.41)		-29.16	-0.20 (-0.25, -0.14)
Ukraine	123.19 (115.08, 131.11)	278.91 (260.56, 296.86)		188.44 (176.03, 201.28)	249.72 (233.28, 266.74)		52.97	-0.46 (-0.62, -0.30)
United Arab Emirates	32.81 (30.04, 35.56)	103.57 (94.81, 112.23)		20.73 (19.19, 22.16)	110.85 (102.62, 118.49)		-36.83	0.30 (0.25, 0.35)
United Kingdom	4.29 (4.00, 4.60)	193.03 (180.16, 207.14)		5.43 (5.08, 5.81)	200.86 (187.95, 214.99)		26.52	0.19 (0.11, 0.27)
Tanzania	57.54 (57.02, 58.09)	150.09 (148.75, 151.52)		53.28 (52.78, 53.76)	140.75 (139.44, 142.01)		-7.4	-0.18 (-0.28, -0.08)
United States of America	443.65 (439.84, 447.46)	221.44 (219.53, 223.34)		442.43 (436.30, 448.41)	241.03 (237.69, 244.29)		-0.28	0.28 (0.08, 0.47)
United States Virgin Islands	0.24 (0.22, 0.25)	219.23 (203.12, 234.88)		0.14 (0.13, 0.14)	224.73 (209.34, 239.44)		-42.74	0.11 (0.08, 0.13)
Uruguay	4.97 (4.69, 5.24)	187.59 (176.99, 198.05)		4.40 (4.14, 4.65)	200.21 (188.51, 211.78)		-11.49	0.26 (0.21, 0.31)
Uzbekistan	42.27 (38.96, 45.54)	123.63 (113.94, 133.20)		40.93 (37.66, 44.42)	119.42 (109.86, 129.60)		-3.16	-0.18 (-0.21, -0.15)
Vanuatu	0.55 (0.51, 0.59)	201.94 (187.92, 215.97)		0.70 (0.66, 0.75)	189.56 (177.38, 202.86)		26.93	-0.20 (-0.24, -0.16)
Venezuela	52.26 (48.98, 55.79)	190.49 (178.53, 203.34)		46.74 (43.63, 49.59)	205.72 (192.04, 218.27)		-10.57	0.35 (0.26, 0.44)
Viet Nam	161.11 (149.45, 173.31)	183.95 (170.65, 197.88)		89.17 (82.14, 96.53)	139.26 (128.28, 150.75)		-44.65	-1.22 (-1.33, -1.11)
Yemen	171.65 (163.47, 179.31)	530.69 (505.41, 554.39)		243.85 (232.09, 256.28)	545.07 (518.79, 572.86)		42.06	0.04 (-0.01, 0.09)
Zambia	48.26 (45.69, 51.23)	263.36 (249.34, 279.54)		73.48 (68.48, 78.40)	250.25 (233.21, 267.00)		52.25	-0.11 (-0.22, 0.01)
Zimbabwe	46.77 (44.24, 49.70)	255.35 (241.52, 271.37)		49.84 (46.95, 53.00)	234.51 (220.90, 249.38)		6.57	-0.36 (-0.67, -0.05)

ASIR: age-standardized rate; CI: confidence interval; EAPC: estimated annual percentage change; GBD: Global Burden of Disease; UI: uncertainty interval.

eTable 3. The deaths and ASMRs of neonatal preterm birth in 1990 and 2019 and their change trends from 1990 to 2019 at national level.

© 2022 Cao G et al. *JAMA Pediatrics.*

Nation	1990		2019		1990-2019	
	Death No. (95% UI)	ASMR per 100,000 No. (95% UI)	Death No. (95% UI)	ASMR per 100,000 No. (95% UI)	Percentage of relative change in death (%)	EAPC of ASMR No. (95% CI)
Afghanistan	6088.26 (3510.81, 9522.36)	24.77 (14.27, 38.80)	8516.31 (4490.35, 13906.54)	12.01 (6.33, 19.61)	39.88	-2.40 (-2.60, -2.20)
Albania	396.70 (327.32, 474.17)	9.63 (7.94, 11.52)	53.19 (33.18, 82.43)	3.16 (1.97, 4.90)	-86.59	-4.23 (-4.62, -3.84)
Algeria	11900.09 (8942.52, 15676.22)	32.30 (24.28, 42.56)	5499.48 (4036.12, 7261.45)	13.09 (9.60, 17.28)	-53.79	-2.96 (-3.11, -2.81)
American Samoa	5.48 (4.11, 6.86)	6.71 (5.03, 8.40)	2.04 (1.45, 2.78)	4.03 (2.85, 5.49)	-62.77	-1.79 (-1.94, -1.63)
Andorra	0.67 (0.51, 0.87)	2.66 (2.01, 3.46)	0.19 (0.13, 0.26)	0.83 (0.57, 1.17)	-71.95	-3.38 (-3.88, -2.87)
Angola	5566.72 (3724.03, 7687.99)	23.01 (15.39, 31.76)	7084.75 (4992.68, 9446.67)	13.38 (9.43, 17.84)	27.27	-1.79 (-1.91, -1.66)
Antigua and Barbuda	6.07 (4.91, 7.44)	9.31 (7.53, 11.41)	2.45 (1.69, 3.34)	5.11 (3.53, 6.98)	-59.71	-2.81 (-3.21, -2.40)
Argentina	5904.31 (5388.54, 6403.79)	17.76 (16.21, 19.27)	1895.09 (1456.98, 2409.66)	5.68 (4.37, 7.22)	-67.9	-3.75 (-3.90, -3.61)
Armenia	751.03 (601.38, 915.69)	20.59 (16.49, 25.11)	75.32 (55.57, 99.42)	4.16 (3.07, 5.49)	-89.97	-6.07 (-6.47, -5.66)
Australia	523.48 (479.57, 576.76)	4.23 (3.88, 4.66)	191.94 (154.35, 232.59)	1.29 (1.04, 1.57)	-63.33	-3.43 (-3.87, -2.99)
Austria	172.89 (152.58, 196.14)	3.86 (3.41, 4.38)	54.63 (44.72, 65.69)	1.28 (1.05, 1.54)	-68.4	-4.18 (-4.76, -3.60)
Azerbaijan	1430.36 (1028.08, 1867.39)	15.73 (11.31, 20.53)	850.84 (618.29, 1132.06)	12.35 (8.98, 16.42)	-40.52	-0.57 (-1.21, 0.07)
Bahamas	22.83 (18.39, 28.10)	8.83 (7.12, 10.87)	6.99 (4.93, 9.46)	3.50 (2.47, 4.73)	-69.37	-2.45 (-2.82, -2.08)
Bahrain	108.71 (90.72, 130.15)	16.55 (13.81, 19.82)	21.20 (16.49, 27.40)	3.34 (2.60, 4.32)	-80.49	-4.87 (-5.58, -4.15)
Bangladesh	62567.93 (51067.99, 75024.24)	33.36 (27.24, 40.00)	7536.37 (4248.88, 12071.75)	5.85 (3.30, 9.37)	-87.95	-6.34 (-6.57, -6.11)
Barbados	18.81 (15.11, 23.26)	9.35 (7.51, 11.56)	7.36 (4.91, 10.64)	5.35 (3.57, 7.73)	-60.88	-1.90 (-2.04, -1.77)
Belarus	297.65 (229.71, 371.91)	4.38 (3.38, 5.48)	41.32 (28.39, 57.33)	0.83 (0.57, 1.15)	-86.12	-6.22 (-6.67, -5.77)
Belgium	240.26 (214.49, 276.41)	3.98 (3.55, 4.58)	58.47 (44.92, 73.75)	0.99 (0.76, 1.24)	-75.67	-4.52 (-5.19, -3.84)
Belize	49.07 (41.17, 58.39)	17.38 (14.58, 20.68)	25.15 (19.33, 31.56)	6.84 (5.25, 8.58)	-48.75	-3.32 (-3.54, -3.09)
Benin	2923.37 (1955.38, 4023.52)	25.72 (17.21, 35.41)	4711.74 (3139.94, 6444.34)	19.57 (13.05, 26.76)	61.18	-0.73 (-0.80, -0.66)
Bermuda	1.49 (1.18, 1.88)	3.50 (2.76, 4.41)	0.24 (0.16, 0.35)	0.98 (0.65, 1.42)	-83.94	-3.62 (-4.45, -2.78)
Bhutan	382.69 (275.87, 505.72)	36.39 (26.22, 48.13)	83.65 (55.18, 120.41)	13.01 (8.58, 18.73)	-78.14	-3.47 (-3.58, -3.36)
Bolivia	3318.09 (2637.93, 4088.12)	28.65 (22.78, 35.33)	1615.43 (1129.94, 2172.11)	10.45 (7.30, 14.05)	-51.31	-3.35 (-3.50, -3.20)
Bosnia and Herzegovina	497.21 (400.10, 631.52)	15.20 (12.23, 19.31)	46.55 (36.32, 57.98)	3.59 (2.80, 4.47)	-90.64	-5.44 (-6.01, -4.87)
Botswana	254.64 (171.76, 352.61)	13.02 (8.78, 18.03)	292.25 (190.97, 436.06)	12.65 (8.26, 18.88)	14.77	0.01 (-0.26, 0.29)
Brazil	46221.39 (40124.64, 53515.69)	28.04 (24.35, 32.47)	11041.82 (8789.55, 13621.88)	7.41 (5.90, 9.15)	-76.11	-4.49 (-4.60, -4.39)
Brunei	12.98 (10.28, 15.80)	3.84 (3.04, 4.67)	10.98 (8.00, 14.84)	3.58 (2.60, 4.84)	-15.39	0.30 (0.02, 0.57)

© 2022 Cao G et al. *JAMA Pediatrics.*

Bulgaria	221.39 (188.10, 268.79)	4.66 (3.96, 5.66)		86.49 (63.84, 111.98)	2.98 (2.20, 3.86)		-60.93	-1.33 (-1.75, -0.92)
Burkina Faso	3482.66 (2348.95, 4843.08)	15.89 (10.72, 22.08)		6142.75 (4087.34, 8727.30)	13.69 (9.11, 19.45)		76.38	-0.09 (-0.34, 0.16)
Burundi	2515.94 (1822.55, 3253.95)	19.98 (14.49, 25.83)		2244.85 (1479.51, 3178.43)	10.08 (6.65, 14.27)		-10.78	-2.38 (-2.49, -2.26)
Cambodia	5025.96 (3859.72, 6383.31)	24.07 (18.48, 30.61)		2244.18 (1563.16, 3128.54)	12.78 (8.90, 17.81)		-55.35	-2.04 (-2.22, -1.86)
Cameroon	4161.78 (2895.02, 5641.17)	18.72 (13.03, 25.37)		5822.80 (4010.12, 7891.80)	13.48 (9.29, 18.28)		39.91	-1.08 (-1.17, -0.99)
Canada	761.05 (693.65, 852.68)	3.89 (3.55, 4.36)		438.75 (373.50, 511.25)	2.42 (2.06, 2.82)		-42.35	-1.05 (-1.27, -0.83)
Cape Verde	82.07 (63.88, 99.65)	14.55 (11.33, 17.66)		39.47 (26.21, 56.96)	7.66 (5.09, 11.06)		-51.91	-1.90 (-2.59, -1.22)
Central African Republic	1591.87 (1130.76, 2160.65)	26.47 (18.83, 35.88)		2209.69 (1498.77, 3096.23)	23.51 (15.95, 32.92)		38.81	-0.07 (-0.23, 0.08)
Chad	3260.91 (2247.94, 4578.49)	21.56 (14.86, 30.33)		6139.86 (3802.10, 8628.95)	16.30 (10.10, 22.90)		88.29	-0.81 (-0.94, -0.67)
Chile	1143.92 (1046.24, 1239.26)	7.99 (7.31, 8.66)		346.32 (249.54, 466.61)	3.17 (2.29, 4.28)		-69.73	-2.44 (-2.74, -2.14)
China	157281.24 (137749.62, 178187.94)	13.63 (11.94, 15.44)		22133.87 (18920.51, 25726.19)	3.07 (2.62, 3.56)		-85.93	-5.36 (-5.64, -5.09)
Colombia	6801.25 (5870.45, 7789.80)	15.21 (13.13, 17.42)		1756.21 (1129.70, 2573.84)	4.61 (2.96, 6.76)		-74.18	-4.23 (-4.56, -3.90)
Comoros	231.90 (155.57, 325.27)	24.66 (16.55, 34.61)		105.88 (60.19, 169.02)	13.39 (7.61, 21.37)		-54.34	-2.04 (-2.22, -1.85)
Congo	727.62 (499.46, 987.23)	15.24 (10.47, 20.67)		784.60 (549.42, 1045.33)	11.39 (7.98, 15.18)		7.83	-0.75 (-0.98, -0.53)
Cook Islands	1.79 (1.30, 2.42)	7.74 (5.62, 10.47)		0.05 (0.02, 0.08)	0.39 (0.18, 0.64)		-97.15	-10.23 (-11.01, -9.45)
Costa Rica	345.62 (289.36, 404.08)	8.87 (7.42, 10.36)		136.19 (92.91, 188.53)	4.26 (2.91, 5.89)		-60.6	-2.31 (-2.50, -2.12)
Cote d'Ivoire	6150.44 (4345.58, 8021.56)	22.61 (15.97, 29.48)		8456.74 (5675.22, 11626.88)	20.03 (13.44, 27.53)		37.5	-0.27 (-0.56, 0.02)
Croatia	148.42 (133.97, 162.69)	5.52 (4.99, 6.06)		15.84 (11.05, 21.71)	0.92 (0.64, 1.26)		-89.33	-6.72 (-7.18, -6.26)
Cuba	355.19 (297.13, 423.33)	4.07 (3.40, 4.85)		50.70 (34.08, 67.37)	1.00 (0.67, 1.33)		-85.73	-4.53 (-5.11, -3.95)
Cyprus	52.01 (42.64, 67.55)	7.84 (6.43, 10.19)		9.72 (6.99, 13.22)	1.32 (0.95, 1.80)		-81.31	-6.22 (-6.69, -5.75)
Czech Republic	458.98 (420.63, 510.91)	7.44 (6.81, 8.28)		47.91 (34.44, 63.93)	0.90 (0.65, 1.20)		-89.56	-7.40 (-8.72, -6.06)
Democratic Republic of the Congo	17331.13 (11985.85, 23508.59)	19.43 (13.45, 26.31)		17225.81 (11234.23, 24199.28)	12.33 (8.04, 17.32)		-0.61	-1.22 (-1.42, -1.03)
Denmark	119.07 (104.37, 134.73)	3.89 (3.41, 4.40)		76.13 (56.63, 97.21)	2.49 (1.86, 3.19)		-36.06	-0.55 (-0.91, -0.18)
Djibouti	168.57 (118.63, 229.73)	15.99 (11.25, 21.75)		168.08 (106.16, 250.48)	10.16 (6.41, 15.13)		-0.29	-1.65 (-1.80, -1.49)
Dominica	6.28 (4.49, 8.20)	7.65 (5.47, 10.00)		3.47 (2.23, 5.06)	8.68 (5.58, 12.66)		-44.73	0.29 (0.07, 0.50)
Dominican Republic	2555.90 (2054.81, 3123.25)	22.36 (17.97, 27.32)		1549.01 (1025.90, 2173.15)	14.16 (9.37, 19.86)		-39.39	-1.19 (-1.38, -0.99)
Ecuador	1961.48 (1610.06, 2360.75)	13.66 (11.22, 16.45)		1124.40 (761.75, 1588.09)	6.69 (4.53, 9.45)		-42.68	-2.67 (-3.25, -2.09)
Egypt	11153.49 (5988.53, 17659.73)	12.34 (6.63, 19.55)		1589.61 (646.56, 2982.17)	1.56 (0.63, 2.93)		-85.75	-7.40 (-8.26, -6.52)
El Salvador	1615.79 (1273.39, 2023.52)	20.59 (16.24, 25.78)		269.00 (180.89, 381.55)	4.96 (3.34, 7.03)		-83.35	-3.99 (-4.35, -3.63)
Equatorial Guinea	198.66 (116.64, 293.06)	18.20 (10.72, 26.86)		188.72 (113.82, 286.76)	10.19 (6.15, 15.48)		-5	-1.98 (-2.26, -1.69)

© 2022 Cao G et al. *JAMA Pediatrics.*

Eritrea	855.39 (593.89, 1177.75)	14.43 (10.02, 19.87)		777.24 (454.28, 1227.18)	8.16 (4.77, 12.88)		-9.14	-1.45 (-1.58, -1.32)
Estonia	29.12 (22.96, 35.77)	2.81 (2.21, 3.45)		3.32 (2.19, 4.37)	0.51 (0.34, 0.68)		-88.6	-5.78 (-6.41, -5.13)
Eswatini	193.59 (136.84, 263.89)	12.70 (8.99, 17.32)		147.11 (102.73, 201.17)	10.46 (7.30, 14.30)		-24.01	-0.43 (-0.61, -0.25)
Ethiopia	26028.25 (20416.08, 32435.95)	22.14 (17.37, 27.58)		16002.76 (11438.12, 21614.24)	9.15 (6.54, 12.36)		-38.52	-3.18 (-3.37, -3.00)
Federated States of Micronesia	22.98 (17.20, 29.54)	13.78 (10.31, 17.72)		4.50 (3.09, 6.18)	4.80 (3.30, 6.60)		-80.41	-3.78 (-3.92, -3.65)
Fiji	90.47 (71.83, 112.40)	9.91 (7.87, 12.31)		78.03 (54.99, 107.89)	9.26 (6.52, 12.79)		-13.75	-0.04 (-0.19, 0.11)
Finland	92.36 (79.16, 106.45)	2.90 (2.49, 3.35)		15.63 (10.75, 19.89)	0.64 (0.44, 0.82)		-83.07	-5.03 (-5.33, -4.73)
France	969.32 (850.40, 1103.56)	2.62 (2.30, 2.99)		318.89 (247.30, 399.44)	0.92 (0.72, 1.16)		-67.1	-3.21 (-3.73, -2.69)
Gabon	326.55 (230.50, 429.59)	17.39 (12.28, 22.87)		205.82 (130.58, 308.72)	10.08 (6.40, 15.13)		-36.97	-1.56 (-1.88, -1.23)
Gambia	408.49 (223.02, 633.99)	17.78 (9.71, 27.60)		346.99 (192.17, 572.69)	10.19 (5.65, 16.82)		-15.06	-1.66 (-1.88, -1.43)
Georgia	511.43 (394.33, 683.56)	12.72 (9.80, 17.01)		65.64 (46.30, 88.24)	2.97 (2.10, 4.00)		-87.17	-5.63 (-6.26, -4.99)
Germany	1894.35 (1659.65, 2152.65)	4.39 (3.84, 4.99)		630.92 (541.57, 716.73)	1.71 (1.47, 1.94)		-66.69	-2.72 (-2.94, -2.51)
Ghana	3024.32 (1697.98, 4893.47)	10.49 (5.89, 16.97)		2712.66 (1510.13, 4358.71)	6.65 (3.70, 10.68)		-10.31	-2.27 (-2.98, -1.55)
Greece	375.96 (326.89, 459.79)	7.51 (6.53, 9.18)		96.65 (75.61, 119.94)	2.32 (1.82, 2.88)		-74.29	-4.20 (-4.79, -3.61)
Greenland	15.31 (13.05, 17.97)	26.54 (22.62, 31.14)		2.65 (1.67, 3.98)	6.86 (4.34, 10.31)		-82.71	-4.71 (-4.95, -4.46)
Grenada	11.01 (8.40, 14.03)	11.54 (8.81, 14.71)		4.30 (2.97, 6.22)	6.44 (4.45, 9.32)		-60.93	-1.85 (-2.23, -1.47)
Guam	8.97 (6.88, 11.27)	4.97 (3.81, 6.24)		11.54 (8.68, 14.95)	7.36 (5.54, 9.54)		28.63	1.93 (1.45, 2.41)
Guatemala	5413.89 (4694.01, 6245.18)	33.60 (29.13, 38.78)		1161.48 (802.05, 1601.31)	5.86 (4.05, 8.09)		-78.55	-6.78 (-7.29, -6.28)
Guinea	4160.58 (3001.25, 5545.34)	27.73 (20.01, 37.00)		3632.23 (2433.21, 5004.13)	15.88 (10.66, 21.87)		-12.7	-2.19 (-2.31, -2.07)
Guinea-Bissau	657.21 (470.25, 877.96)	29.63 (21.23, 39.51)		555.25 (387.26, 746.87)	18.63 (12.99, 25.05)		-15.51	-1.42 (-1.59, -1.24)
Guyana	330.00 (271.90, 398.10)	31.05 (25.58, 37.47)		86.60 (58.51, 124.00)	12.61 (8.52, 18.06)		-73.76	-2.85 (-3.05, -2.66)
Haiti	1232.55 (714.54, 1925.39)	10.48 (6.08, 16.37)		1556.92 (861.50, 2387.89)	9.85 (5.45, 15.10)		26.32	0.02 (-0.08, 0.11)
Honduras	1523.35 (1184.00, 1843.52)	17.73 (13.78, 21.47)		799.78 (559.94, 1073.65)	7.09 (4.96, 9.52)		-47.5	-3.02 (-3.34, -2.71)
Hungary	574.70 (505.07, 630.21)	9.64 (8.47, 10.57)		64.36 (42.83, 88.71)	1.61 (1.07, 2.22)		-88.8	-6.17 (-6.44, -5.89)
Iceland	9.37 (8.10, 10.94)	4.20 (3.63, 4.90)		2.39 (1.44, 3.86)	1.16 (0.70, 1.88)		-74.48	-4.65 (-5.10, -4.19)
India	317674.34 (273312.82, 371644.24)	25.32 (21.78, 29.63)		165604.31 (132584.48, 204832.30)	14.71 (11.78, 18.19)		-47.87	-1.44 (-1.64, -1.24)
Indonesia	49805.25 (41417.36, 58517.78)	21.84 (18.16, 25.66)		17792.35 (13709.27, 22707.78)	9.65 (7.44, 12.32)		-64.28	-2.81 (-2.94, -2.68)
Iran	32989.75 (27058.25, 40162.45)	38.54 (31.61, 46.92)		4459.08 (3545.97, 5496.22)	6.86 (5.46, 8.46)		-86.48	-5.14 (-5.73, -4.54)
Iraq	11661.24 (9871.94, 13666.95)	34.76 (29.44, 40.73)		4553.32 (3194.62, 6401.92)	9.89 (6.94, 13.91)		-60.95	-3.92 (-4.36, -3.47)
Ireland	108.37 (97.32, 123.02)	4.19 (3.76, 4.75)		40.48 (30.17, 52.87)	1.39 (1.03, 1.81)		-62.65	-3.93 (-4.28, -3.58)

© 2022 Cao G et al. *JAMA Pediatrics.*

Israel	357.56 (320.69, 412.38)	7.14 (6.40, 8.23)		96.29 (70.96, 125.64)	1.03 (0.76, 1.34)		-73.07	-6.60 (-6.76, -6.43)
Italy	1573.46 (1454.92, 1714.05)	5.85 (5.40, 6.37)		254.89 (206.75, 301.45)	1.19 (0.96, 1.40)		-83.8	-5.63 (-5.93, -5.33)
Jamaica	508.26 (421.90, 604.74)	18.87 (15.66, 22.45)		230.32 (160.46, 324.67)	13.28 (9.26, 18.73)		-54.68	-1.51 (-1.70, -1.32)
Japan	1061.37 (989.49, 1149.47)	1.78 (1.66, 1.93)		181.10 (146.29, 210.60)	0.41 (0.33, 0.48)		-82.94	-4.81 (-5.08, -4.53)
Jordan	1312.01 (1072.19, 1590.99)	20.05 (16.39, 24.32)		1009.63 (727.08, 1425.54)	8.54 (6.15, 12.06)		-23.05	-3.12 (-3.34, -2.90)
Kazakhstan	1801.80 (1495.06, 2157.28)	10.54 (8.74, 12.61)		789.00 (561.05, 1039.17)	4.63 (3.29, 6.10)		-56.21	-2.94 (-3.81, -2.07)
Kenya	4831.96 (3676.21, 6045.46)	10.11 (7.69, 12.64)		4362.44 (3422.40, 5538.71)	6.78 (5.32, 8.61)		-9.72	-1.29 (-1.44, -1.14)
Kiribati	33.80 (25.26, 44.07)	27.55 (20.58, 35.92)		22.46 (15.35, 31.69)	15.49 (10.59, 21.86)		-33.54	-1.89 (-1.94, -1.85)
Kuwait	188.20 (159.50, 227.29)	10.17 (8.62, 12.28)		172.53 (131.80, 223.32)	5.84 (4.47, 7.57)		-8.32	-0.08 (-0.66, 0.51)
Kyrgyzstan	950.54 (730.57, 1216.28)	15.72 (12.08, 20.11)		595.63 (498.78, 702.11)	8.53 (7.14, 10.05)		-37.34	-0.93 (-1.70, -0.15)
Lao	3613.46 (2852.03, 4486.35)	45.87 (36.07, 57.00)		1518.31 (1123.96, 1958.18)	18.51 (13.70, 23.86)		-57.98	-3.18 (-3.39, -2.98)
Latvia	35.71 (28.66, 43.70)	2.02 (1.62, 2.48)		3.96 (2.58, 5.32)	0.43 (0.28, 0.57)		-88.91	-6.21 (-6.73, -5.69)
Lebanon	1123.74 (897.85, 1365.59)	21.36 (17.06, 25.96)		240.24 (135.18, 366.77)	4.73 (2.66, 7.22)		-78.62	-5.08 (-5.18, -4.97)
Lesotho	676.61 (449.59, 927.15)	23.39 (15.56, 32.05)		404.25 (258.72, 583.25)	18.25 (11.68, 26.33)		-40.25	-0.82 (-0.99, -0.66)
Liberia	1184.65 (859.80, 1507.53)	30.95 (22.47, 39.36)		779.09 (501.48, 1149.64)	12.12 (7.80, 17.88)		-34.23	-2.94 (-3.15, -2.73)
Libya	1823.40 (1422.07, 2330.16)	25.37 (19.79, 32.42)		174.98 (123.79, 240.57)	4.44 (3.15, 6.11)		-90.4	-6.24 (-6.43, -6.05)
Lithuania	119.31 (101.12, 138.26)	4.42 (3.74, 5.12)		9.74 (6.65, 12.48)	0.75 (0.51, 0.96)		-91.84	-6.96 (-7.46, -6.45)
Luxembourg	6.94 (5.86, 8.31)	2.90 (2.45, 3.47)		1.63 (0.95, 2.65)	0.51 (0.30, 0.83)		-76.49	-5.94 (-6.49, -5.37)
Macedonia	362.09 (325.16, 401.76)	21.48 (19.28, 23.83)		82.04 (66.44, 99.63)	7.66 (6.20, 9.30)		-77.34	-2.55 (-3.09, -2.02)
Madagascar	5119.27 (3903.78, 6532.83)	20.19 (15.38, 25.77)		5122.04 (3455.82, 7217.55)	12.51 (8.44, 17.62)		0.05	-1.36 (-1.45, -1.28)
Malawi	4123.30 (3029.60, 5492.25)	18.64 (13.69, 24.79)		2587.81 (1617.68, 3756.47)	9.86 (6.17, 14.31)		-37.24	-2.18 (-2.27, -2.09)
Malaysia	1592.42 (1234.81, 2082.69)	6.67 (5.17, 8.72)		524.53 (386.42, 685.25)	2.01 (1.48, 2.62)		-67.06	-3.59 (-4.37, -2.81)
Maldives	111.64 (84.85, 144.87)	26.52 (20.16, 34.42)		25.98 (17.48, 37.04)	6.40 (4.30, 9.13)		-76.73	-4.87 (-5.03, -4.71)
Mali	7175.10 (5148.97, 9814.01)	34.03 (24.42, 46.59)		10624.37 (7253.94, 14729.59)	23.10 (15.79, 32.04)		48.07	-1.22 (-1.46, -0.99)
Malta	21.75 (19.16, 25.37)	8.04 (7.08, 9.38)		7.41 (4.82, 10.97)	3.57 (2.33, 5.29)		-65.93	-2.72 (-3.12, -2.32)
Marshall Islands	10.02 (7.87, 12.34)	13.74 (10.78, 16.93)		5.09 (3.68, 6.94)	8.59 (6.20, 11.69)		-49.16	-1.72 (-2.18, -1.26)
Mauritania	965.53 (697.57, 1267.57)	22.66 (16.38, 29.72)		672.19 (462.39, 920.85)	12.82 (8.82, 17.56)		-30.38	-1.69 (-1.81, -1.57)
Mauritius	195.80 (181.57, 211.56)	18.14 (16.82, 19.60)		43.53 (31.32, 58.33)	7.04 (5.06, 9.43)		-77.77	-3.77 (-4.19, -3.35)
Mexico	18374.91 (14612.81, 22586.17)	15.43 (12.27, 18.97)		5374.61 (4050.80, 6787.75)	5.33 (4.01, 6.73)		-70.75	-3.55 (-3.74, -3.36)
Moldova	142.25 (101.91, 192.26)	3.82 (2.73, 5.17)		33.60 (24.29, 45.42)	2.17 (1.57, 2.93)		-76.38	-1.44 (-2.20, -0.68)

© 2022 Cao G et al. *JAMA Pediatrics*.

Monaco	0.23 (0.15, 0.34)	1.78 (1.16, 2.61)		0.08 (0.05, 0.11)	0.49 (0.33, 0.69)		-67.96	-4.33 (-4.42, -4.24)
Mongolia	500.05 (369.19, 659.51)	14.31 (10.57, 18.87)		200.26 (136.87, 271.40)	5.20 (3.55, 7.04)		-59.95	-3.44 (-3.99, -2.89)
Montenegro	44.34 (37.35, 52.26)	9.21 (7.75, 10.86)		4.65 (2.84, 6.09)	1.44 (0.88, 1.88)		-89.52	-6.69 (-7.43, -5.94)
Morocco	11999.24 (9342.52, 15098.30)	31.72 (24.69, 39.91)		2858.64 (1975.78, 3979.92)	9.90 (6.84, 13.77)		-76.18	-3.74 (-4.07, -3.41)
Mozambique	5528.48 (3754.66, 7578.16)	19.88 (13.53, 27.25)		6223.58 (4119.17, 8736.99)	11.59 (7.67, 16.26)		12.57	-1.81 (-1.95, -1.66)
Myanmar	18267.32 (12487.77, 25583.55)	28.86 (19.75, 40.44)		7368.27 (4909.43, 10218.97)	14.73 (9.82, 20.43)		-59.66	-2.39 (-2.62, -2.16)
Namibia	322.83 (223.68, 428.05)	13.93 (9.65, 18.47)		279.01 (185.29, 414.94)	9.31 (6.18, 13.85)		-13.57	-0.96 (-1.12, -0.80)
Nauru	2.33 (1.72, 3.04)	11.70 (8.64, 15.23)		1.44 (1.07, 1.90)	10.11 (7.48, 13.31)		-38.23	0.02 (-0.68, 0.72)
Nepal	3873.49 (2390.82, 5965.69)	9.96 (6.16, 15.33)		798.37 (463.47, 1266.91)	2.71 (1.57, 4.30)		-79.39	-4.96 (-5.35, -4.57)
Netherlands	344.93 (307.51, 383.65)	3.62 (3.22, 4.02)		125.20 (95.31, 159.88)	1.45 (1.10, 1.85)		-63.7	-3.17 (-3.49, -2.85)
New Zealand	128.74 (118.32, 140.85)	4.47 (4.10, 4.89)		59.98 (45.88, 75.86)	2.09 (1.60, 2.65)		-53.41	-1.60 (-2.09, -1.10)
Nicaragua	1274.62 (1075.79, 1489.02)	18.51 (15.63, 21.63)		272.98 (202.89, 353.02)	4.33 (3.22, 5.60)		-78.58	-4.75 (-4.84, -4.65)
Niger	3790.22 (2674.97, 5018.45)	17.93 (12.68, 23.71)		7789.72 (5566.01, 10358.18)	14.20 (10.16, 18.88)		105.52	-1.21 (-1.43, -1.00)
Nigeria	42886.39 (32659.01, 52574.34)	21.75 (16.56, 26.68)		76403.66 (58066.76, 98959.59)	21.20 (16.11, 27.45)		78.15	0.12 (0.02, 0.22)
Niue	0.25 (0.18, 0.32)	9.46 (6.83, 12.42)		0.09 (0.06, 0.12)	7.14 (5.07, 9.45)		-64.59	-1.16 (-1.46, -0.87)
North Korea	7890.47 (5771.41, 10467.07)	22.98 (16.81, 30.48)		992.16 (722.66, 1351.24)	6.01 (4.38, 8.18)		-87.43	-4.27 (-4.63, -3.90)
Northern Mariana Islands	3.02 (2.13, 4.09)	4.96 (3.51, 6.72)		1.11 (0.83, 1.42)	4.71 (3.53, 6.01)		-63.27	0.80 (0.03, 1.59)
Norway	60.48 (51.78, 67.63)	2.08 (1.78, 2.33)		14.30 (10.32, 17.76)	0.51 (0.37, 0.64)		-76.35	-4.02 (-4.36, -3.68)
Oman	612.26 (450.16, 816.43)	19.29 (14.19, 25.72)		188.58 (146.45, 238.24)	5.01 (3.89, 6.34)		-69.2	-4.15 (-5.27, -3.01)
Pakistan	54102.51 (41066.99, 68850.02)	23.56 (17.87, 29.98)		59399.79 (42951.20, 77884.25)	18.69 (13.52, 24.51)		9.79	-0.61 (-0.70, -0.51)
Palau	2.27 (1.70, 2.92)	12.84 (9.61, 16.53)		0.47 (0.32, 0.65)	5.15 (3.53, 7.16)		-79.47	-2.68 (-2.87, -2.49)
Palestine	1257.89 (1048.78, 1503.59)	27.73 (23.11, 33.13)		469.87 (353.32, 629.13)	7.80 (5.87, 10.45)		-62.65	-3.80 (-4.30, -3.30)
Panama	278.37 (229.71, 334.87)	9.80 (8.09, 11.79)		165.35 (114.56, 227.76)	4.49 (3.11, 6.19)		-40.6	-2.87 (-3.17, -2.57)
Papua New Guinea	1057.72 (717.71, 1451.25)	14.89 (10.11, 20.45)		2014.44 (1304.74, 2988.97)	12.88 (8.34, 19.10)		90.45	-0.35 (-0.45, -0.26)
Paraguay	796.76 (660.05, 951.22)	12.98 (10.75, 15.50)		267.83 (176.90, 379.17)	4.40 (2.90, 6.23)		-66.38	-3.61 (-4.13, -3.08)
Peru	5648.51 (4449.40, 7134.38)	17.82 (14.04, 22.52)		1339.88 (845.53, 1969.04)	4.36 (2.75, 6.42)		-76.28	-4.53 (-4.71, -4.35)
Philippines	13196.55 (11081.57, 15491.22)	13.32 (11.19, 15.64)		9915.74 (7325.45, 12930.46)	7.80 (5.76, 10.17)		-24.86	-1.53 (-1.67, -1.38)
Poland	2929.27 (2621.14, 3232.18)	11.32 (10.13, 12.50)		385.97 (271.39, 525.14)	2.20 (1.55, 2.99)		-86.82	-5.34 (-5.90, -4.77)
Portugal	400.63 (347.83, 472.47)	7.23 (6.28, 8.53)		32.42 (22.74, 43.09)	0.84 (0.59, 1.12)		-91.91	-7.17 (-7.60, -6.74)
Puerto Rico	400.88 (364.08, 438.22)	12.69 (11.53, 13.88)		51.58 (37.10, 70.28)	4.23 (3.05, 5.77)		-87.13	-3.92 (-4.30, -3.55)

© 2022 Cao G et al. *JAMA Pediatrics.*

Qatar	100.27 (77.92, 126.45)	19.84 (15.42, 25.01)		65.64 (49.56, 86.97)	4.98 (3.76, 6.60)		-34.54	-5.06 (-5.22, -4.90)
Romania	608.74 (491.14, 743.37)	4.24 (3.42, 5.19)		222.60 (168.60, 284.78)	2.65 (2.01, 3.40)		-63.43	-1.45 (-1.76, -1.14)
Russia	5856.75 (5489.59, 6545.39)	6.27 (5.88, 7.01)		1064.68 (827.06, 1336.72)	1.32 (1.02, 1.66)		-81.82	-5.67 (-6.13, -5.21)
Rwanda	3082.43 (2218.64, 4145.06)	20.33 (14.63, 27.33)		1444.57 (948.52, 2019.00)	8.56 (5.62, 11.96)		-53.14	-2.85 (-3.19, -2.52)
Saint Kitts and Nevis	7.03 (5.92, 8.42)	16.20 (13.64, 19.41)		2.29 (1.72, 2.95)	6.83 (5.12, 8.79)		-67.36	-2.90 (-3.30, -2.51)
Saint Lucia	26.36 (21.27, 32.22)	14.51 (11.71, 17.74)		8.06 (5.43, 11.47)	9.42 (6.35, 13.41)		-69.41	-1.21 (-1.37, -1.05)
Saint Vincent and the Grenadines	23.87 (18.36, 30.08)	18.73 (14.41, 23.61)		5.98 (4.05, 8.40)	8.21 (5.57, 11.54)		-74.94	-2.72 (-2.93, -2.52)
Samoa	21.79 (15.17, 30.69)	13.16 (9.15, 18.52)		7.77 (4.86, 11.30)	4.21 (2.63, 6.13)		-64.36	-3.64 (-3.79, -3.50)
San Marino	0.31 (0.20, 0.46)	2.36 (1.53, 3.43)		0.13 (0.08, 0.19)	0.86 (0.54, 1.25)		-57.94	-3.59 (-3.84, -3.34)
Sao Tome and Principe	19.15 (12.74, 25.94)	8.62 (5.74, 11.66)		12.53 (7.17, 18.90)	5.57 (3.19, 8.41)		-34.61	-1.21 (-1.44, -0.97)
Saudi Arabia	6366.05 (4650.68, 8330.31)	24.80 (18.12, 32.45)		518.14 (361.72, 708.99)	2.36 (1.64, 3.22)		-91.86	-7.87 (-8.30, -7.43)
Senegal	3062.33 (2221.18, 4015.88)	18.74 (13.59, 24.58)		3157.93 (2094.69, 4337.09)	14.23 (9.44, 19.54)		3.12	-0.91 (-1.04, -0.77)
Serbia	1143.55 (1003.56, 1311.92)	17.87 (15.68, 20.50)		104.93 (79.24, 133.23)	2.63 (1.99, 3.34)		-90.82	-6.34 (-6.96, -5.71)
Seychelles	8.72 (6.72, 10.83)	10.77 (8.30, 13.38)		3.42 (2.36, 4.73)	4.80 (3.31, 6.63)		-60.76	-1.97 (-2.29, -1.64)
Sierra Leone	2503.88 (1755.91, 3461.90)	33.45 (23.47, 46.25)		2325.11 (1603.28, 3259.91)	17.48 (12.05, 24.51)		-7.14	-2.31 (-2.39, -2.23)
Singapore	81.46 (71.71, 97.56)	3.32 (2.93, 3.98)		13.58 (8.86, 19.88)	0.49 (0.32, 0.72)		-83.33	-5.80 (-6.24, -5.36)
Slovakia	355.01 (314.78, 398.25)	9.36 (8.29, 10.50)		82.87 (61.57, 109.49)	3.06 (2.28, 4.04)		-76.66	-3.66 (-3.94, -3.38)
Slovenia	65.06 (57.54, 72.09)	6.08 (5.37, 6.74)		8.58 (5.45, 11.85)	0.94 (0.60, 1.30)		-86.81	-5.84 (-6.15, -5.52)
Solomon Islands	76.09 (46.86, 105.87)	11.14 (6.86, 15.51)		58.84 (34.29, 87.91)	5.89 (3.43, 8.80)		-22.67	-2.22 (-2.44, -2.01)
Somalia	2895.59 (1989.03, 4109.10)	19.76 (13.56, 28.01)		4923.00 (3121.34, 7496.07)	11.88 (7.54, 18.08)		70.02	-1.24 (-1.39, -1.10)
South Africa	5915.38 (4661.16, 7277.67)	12.70 (10.01, 15.63)		6378.64 (4826.03, 8332.67)	12.90 (9.76, 16.85)		7.83	0.90 (0.30, 1.50)
South Korea	1655.02 (1352.50, 2079.98)	5.08 (4.15, 6.40)		285.70 (235.64, 340.88)	1.44 (1.18, 1.71)		-82.74	-3.48 (-4.00, -2.95)
South Sudan	2737.34 (1946.75, 3673.52)	22.96 (16.34, 30.74)		2517.68 (1648.72, 3524.66)	14.61 (9.57, 20.44)		-8.02	-1.26 (-1.42, -1.10)
Spain	884.56 (795.35, 1009.40)	4.62 (4.15, 5.27)		159.31 (125.05, 198.00)	0.89 (0.70, 1.11)		-81.99	-5.23 (-5.63, -4.82)
Sri Lanka	1742.66 (1377.26, 2160.37)	10.16 (8.03, 12.59)		525.92 (348.50, 763.78)	3.68 (2.43, 5.34)		-69.82	-2.75 (-3.25, -2.26)
Sudan	26705.76 (20245.42, 35265.30)	57.98 (43.97, 76.97)		14192.45 (10360.05, 19089.00)	25.24 (18.43, 33.95)		-46.86	-2.41 (-2.57, -2.26)
Suriname	130.48 (107.49, 153.52)	29.98 (24.69, 35.27)		65.03 (43.82, 92.99)	15.10 (10.17, 21.59)		-50.16	-2.51 (-2.64, -2.37)
Sweden	136.32 (121.15, 151.04)	2.27 (2.02, 2.52)		41.33 (29.79, 51.93)	0.72 (0.52, 0.91)		-69.68	-3.49 (-4.10, -2.89)
Switzerland	125.94 (109.50, 145.70)	3.14 (2.73, 3.64)		62.77 (52.40, 75.01)	1.47 (1.23, 1.76)		-50.16	-2.18 (-2.42, -1.95)
Syrian Arab Republic	5624.96 (3905.63, 7593.71)	23.31 (16.19, 31.47)		451.83 (290.51, 641.71)	4.03 (2.59, 5.73)		-91.97	-5.41 (-6.85, -3.95)

© 2022 Cao G et al. *JAMA Pediatrics.*

Taiwan (Province of China)	303.27 (257.83, 344.94)	1.90 (1.61, 2.16)		141.15 (106.72, 184.65)	1.65 (1.25, 2.16)		-53.46	-2.25 (-3.63, -0.85)
Tajikistan	808.56 (560.47, 1102.78)	8.25 (5.72, 11.25)		739.03 (507.83, 1032.98)	6.06 (4.16, 8.47)		-8.6	-1.19 (-1.48, -0.89)
Thailand	8087.51 (5598.67, 11054.48)	14.00 (9.71, 19.10)		10129.58 (6431.99, 15038.42)	10.27 (6.53, 15.25)		25.25	-0.68 (-0.96, -0.40)
Timor-Leste	7424.61 (5735.13, 9252.80)	14.57 (11.26, 18.16)		715.52 (522.54, 951.73)	2.53 (1.85, 3.37)		-90.36	-6.25 (-6.58, -5.92)
Togo	461.48 (362.28, 581.11)	27.18 (21.32, 34.18)		251.97 (178.97, 330.46)	13.71 (9.74, 17.98)		-45.4	-2.34 (-2.45, -2.23)
Tokelau	1774.37 (1256.10, 2315.58)	22.73 (16.11, 29.66)		1619.64 (1058.15, 2300.79)	14.34 (9.37, 20.36)		-8.72	-1.49 (-1.53, -1.46)
Tonga	0.27 (0.20, 0.36)	9.49 (6.99, 12.65)		0.04 (0.02, 0.05)	2.07 (1.30, 2.96)		-86.44	-5.30 (-5.58, -5.03)
Trinidad and Tobago	13.06 (9.80, 16.77)	10.44 (7.84, 13.41)		5.60 (3.67, 8.02)	4.98 (3.26, 7.13)		-57.11	-2.47 (-2.62, -2.31)
Tunisia	166.43 (134.54, 202.00)	14.76 (11.93, 17.92)		53.11 (35.59, 75.98)	7.16 (4.80, 10.25)		-68.09	-3.10 (-3.56, -2.64)
Turkey	3648.46 (2825.70, 4657.87)	32.12 (24.88, 41.01)		507.03 (381.00, 657.89)	6.29 (4.73, 8.16)		-86.1	-5.39 (-5.55, -5.23)
Turkmenistan	30592.92 (23900.59, 37965.10)	38.86 (30.37, 48.22)		3821.89 (2809.88, 4939.85)	8.10 (5.96, 10.47)		-87.51	-5.20 (-5.34, -5.07)
Tuvalu	671.36 (474.59, 916.32)	10.71 (7.58, 14.61)		513.14 (385.55, 679.04)	9.55 (7.17, 12.63)		-23.57	0.27 (-0.33, 0.87)
Uganda	3.50 (2.65, 4.47)	25.39 (19.24, 32.40)		0.54 (0.36, 0.78)	5.20 (3.47, 7.48)		-84.49	-5.39 (-5.48, -5.31)
Ukraine	6413.04 (4219.54, 9186.98)	14.59 (9.62, 20.84)		8359.92 (4716.19, 12675.06)	11.09 (6.26, 16.81)		30.36	-0.58 (-0.75, -0.40)
United Arab Emirates	2071.76 (1495.26, 2771.65)	6.53 (4.71, 8.74)		363.81 (270.86, 473.32)	1.94 (1.44, 2.52)		-82.44	-4.59 (-5.00, -4.17)
United Kingdom	203.59 (142.31, 285.56)	9.14 (6.39, 12.82)		35.48 (21.34, 52.96)	1.30 (0.78, 1.95)		-82.57	-5.65 (-6.13, -5.18)
Tanzania	1955.03 (1769.76, 2082.11)	5.09 (4.61, 5.42)		1103.22 (906.40, 1324.38)	2.90 (2.39, 3.49)		-43.57	-1.79 (-2.01, -1.58)
United States of America	12229.92 (11607.64, 12940.54)	6.09 (5.78, 6.45)		6400.41 (5824.37, 7049.35)	3.48 (3.17, 3.83)		-47.67	-1.65 (-1.77, -1.52)
United States Virgin Islands	12.82 (10.53, 15.27)	11.83 (9.72, 14.10)		2.19 (1.48, 3.10)	3.61 (2.44, 5.12)		-82.92	-3.91 (-4.08, -3.75)
Uruguay	315.43 (272.06, 350.39)	11.90 (10.26, 13.22)		67.73 (47.32, 94.52)	3.08 (2.15, 4.29)		-78.53	-4.98 (-5.28, -4.67)
Uzbekistan	1917.93 (1309.02, 2545.44)	5.61 (3.83, 7.44)		1829.90 (1414.09, 2391.43)	5.33 (4.12, 6.97)		-4.59	0.07 (-0.15, 0.30)
Vanuatu	36.54 (26.41, 48.43)	13.38 (9.67, 17.73)		35.10 (25.36, 46.36)	9.49 (6.86, 12.53)		-3.94	-0.89 (-1.13, -0.65)
Venezuela	4014.66 (3434.50, 4525.07)	14.66 (12.54, 16.52)		1646.29 (1139.83, 2369.93)	7.23 (5.01, 10.42)		-58.99	-2.06 (-2.42, -1.70)
Viet Nam	17772.10 (14138.50, 21571.25)	20.29 (16.14, 24.63)		3556.87 (2300.21, 4987.55)	5.55 (3.59, 7.77)		-79.99	-4.31 (-4.46, -4.16)
Yemen	13208.67 (7218.16, 20502.32)	41.42 (22.62, 64.24)		10248.79 (6626.53, 14623.75)	22.94 (14.83, 32.74)		-22.41	-1.91 (-1.97, -1.86)
Zambia	1945.42 (1349.17, 2599.52)	10.70 (7.44, 14.31)		2195.63 (1345.03, 3271.42)	7.48 (4.58, 11.15)		12.86	-1.04 (-1.32, -0.77)
Zimbabwe	2826.36 (2105.31, 3519.14)	15.44 (11.50, 19.23)		3604.76 (2546.26, 4969.19)	16.97 (11.99, 23.39)		27.54	0.58 (0.40, 0.76)

ASMR: age-standardized mortality rate; CI: confidence interval; EAPC: estimated annual percentage change; UI: uncertainty interval.

© 2022 Cao G et al. *JAMA Pediatrics.*
